# Sound‐guided assessment and localization of pulmonary air leak

**DOI:** 10.1002/btm2.10322

**Published:** 2022-05-04

**Authors:** Meghan R. Pinezich, Seyed Mohammad Mir, Jonathan A. Reimer, Sarah R. Kaslow, Jiawen Chen, Brandon A. Guenthart, Matthew Bacchetta, John D. O'Neill, Gordana Vunjak‐Novakovic, Jinho Kim

**Affiliations:** ^1^ Department of Biomedical Engineering Columbia University New York New York USA; ^2^ Department of Biomedical Engineering Stevens Institute of Technology Hoboken New Jersey USA; ^3^ Department of Surgery Columbia University Medical Center New York New York USA; ^4^ Department of Cardiothoracic Surgery Stanford University Stanford California USA; ^5^ Department of Thoracic Surgery, Vanderbilt University Nashville Tennessee USA; ^6^ Department of Cell Biology State University of New York Downstate Medical Center Brooklyn New York USA; ^7^ Department of Medicine Columbia University Medical Center New York New York USA

**Keywords:** digital medicine, Lung disease, lung volume reduction surgery, minimally‐invasive diagnosis, sound analysis

## Abstract

Pulmonary air leak is the most common complication of lung surgery, with air leaks that persist longer than 5 days representing a major source of post‐surgery morbidity. Clinical management of air leaks is challenging due to limited methods to precisely locate and assess leaks. Here, we present a sound‐guided methodology that enables rapid quantitative assessment and precise localization of air leaks by analyzing the distinct sounds generated as the air escapes through defective lung tissue. Air leaks often present after lung surgery due to loss of tissue integrity at or near a staple line. Accordingly, we investigated air leak sounds from a focal pleural defect in a rat model and from a staple line failure in a clinically relevant swine model to demonstrate the high sensitivity and translational potential of this approach. In rat and swine models of free‐flowing air leak under positive pressure ventilation with intrapleural microphone 1 cm from the lung surface, we identified that: (a) pulmonary air leaks generate sounds that contain distinct harmonic series, (b) acoustic characteristics of air leak sounds can be used to classify leak severity, and (c) precise location of the air leak can be determined with high resolution (within 1 cm) by mapping the sound loudness level across the lung surface. Our findings suggest that sound‐guided assessment and localization of pulmonary air leaks could serve as a diagnostic tool to inform air leak detection and treatment strategies during video‐assisted thoracoscopic surgery (VATS) or thoracotomy procedures.

## 1 INTRODUCTION

Pulmonary air leak is a common complication of lung surgery, occurring in up to 60% of patients undergoing lung resection, frequently due to faulty staple‐lines through compromised tissue.^1^, ^2^, ^3^, ^4^, ^5^, ^6^ Pulmonary air leaks range in severity from “mild leaks” that often resolve spontaneously, to more serious “prolonged leaks” that require up to weeks to heal and often necessitate additional interventions.^7^, ^8^, ^9^ Prolonged air leaks that persist beyond 5 days substantially increase the risk of complications, such as empyema and pneumonia, and are associated with increased hospital length of stay and cost.^8^ Patients with severe air leaks develop pneumothorax, which can rapidly lead to tension pneumothorax, cardiovascular collapse, and death (Figure S1).^10^ Among the diverse population of lung surgery patients, most have underlying lung disease include chronic obstructive pulmonary disease (COPD), interstitial lung disease (ILD), and lung cancer, which predisposes them to severe, prolonged air leaks. In these patients, to quantify and localize air leaks or assess prognosis remains challenging. Methods to objectively assess severity and precisely locate pulmonary air leaks would enable targeted intervention and treatment.

For centuries, clinicians have relied on qualitative auscultation of lung sounds generated by airflow within the respiratory tract to diagnose respiratory conditions.^11^, ^12^ The properties of a periodic acoustic wave (e.g., frequency, amplitude) determine loudness, spectral density, and pitch. Waves with frequency between 20 Hz and 20 kHz are perceived as audible sounds.^13^, ^14^ As fluid passes by a structure, energy is transferred from the fluid to the structure, causing vibration that produces sound with intensities and frequencies dependent on the amplitudes and modes of vibration.^15^ For example, the primary mechanism of human voice production is oscillation of the vocal cords produced by airflow through the glottis (fundamental frequency range: 110–300 Hz).^16^, ^17^ Significantly, we discovered that airflow through defective visceral pleura, that is, pulmonary air leak, also causes oscillations in the surrounding lung tissue, resulting in distinct, quantifiable air leak sounds (Figure S2). This discovery led us to hypothesize that acoustic signatures of these sounds provide quantifiable information about the air leak, such as leak severity and location.

**FIGURE 1:**
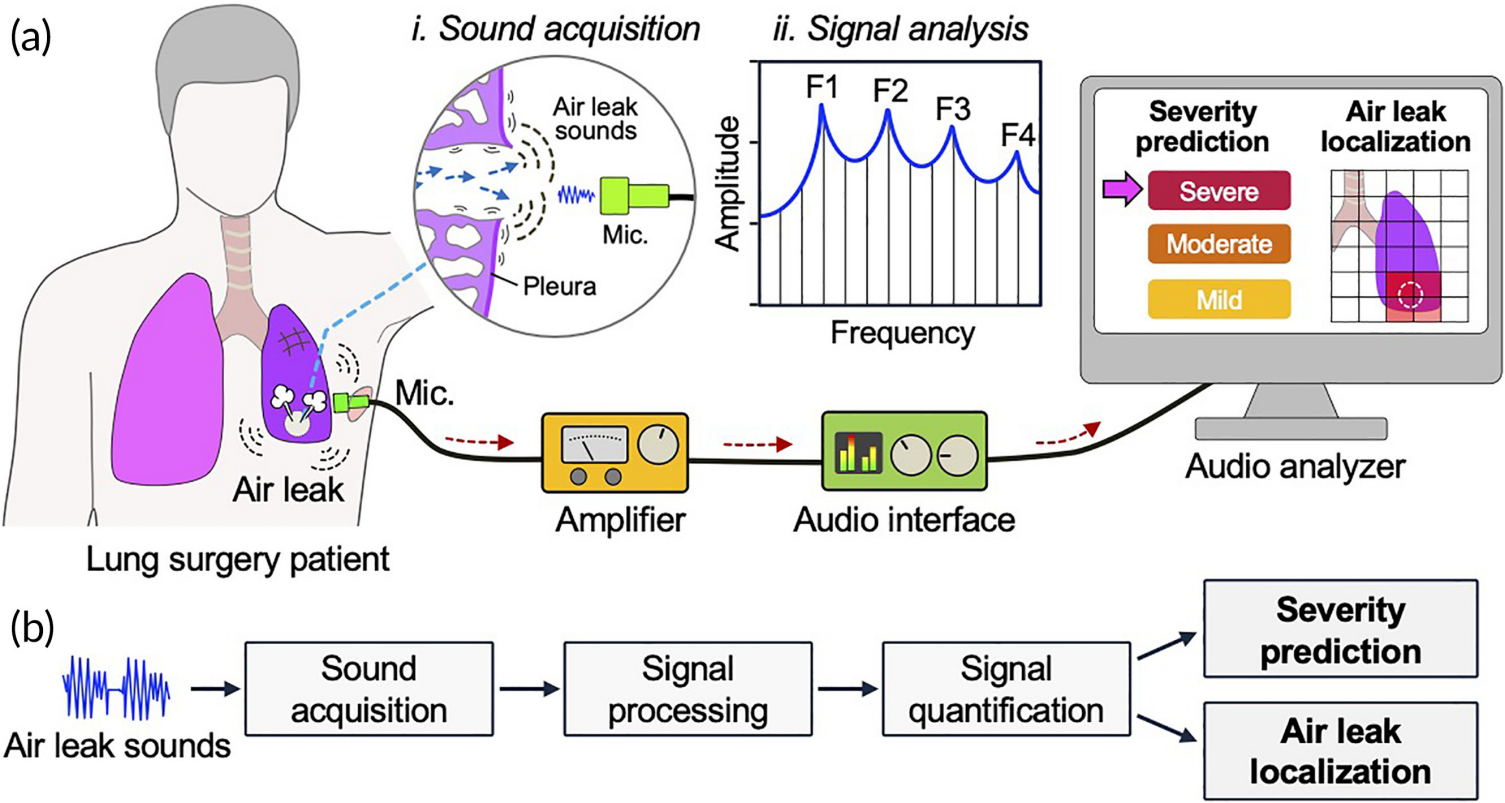
Assessment and localization of pulmonary air leak using sound analysis. (a) Schematic of pulmonary air leak with computer‐assisted sound analysis system. Mic: microphone. F: frequency (b) Process flow diagram of air leak sound analysis for severity prediction and localization to improve clinical decision‐making for patients with air leak

Clinical methods used to detect and assess pulmonary air leak remain limited, and do not precisely assess leak severity or locate leak site. We hypothesized that quantitative analysis of air leak sounds could enable detection, severity assessment, and precise localization of pulmonary air leak. To test this hypothesis, we developed a sound analysis system and methodology to evaluate pulmonary air leak in situ (Figure 1 and Figure S3). In this study, we show that pulmonary air leaks can be evaluated by analyzing air leak sound signals. Further, we demonstrate that air leak sound profiles can be used to classify severity and localize the air leak with significantly higher resolution than any currently available methods. Using a clinically relevant large animal model (swine) of free‐flowing air leak in an open thoracotomy, we evaluated post‐surgical air leaks due to staple‐line failure, and applied our quantitative sound‐guided methodology to distinguish mild and severe air leaks and to precisely localize air leak sites. We identified that air leak sounds contain frequencies that comprise harmonic series, a finding not previously reported. Collectively, our results indicate that pulmonary air leak sounds are quantifiable in a clinically relevant setting, and could be used for real‐time severity assessment and precise localization of air leaks.

## 2 RESULTS

### 2.1 Rat model of pulmonary air leak

**FIGURE 2:**
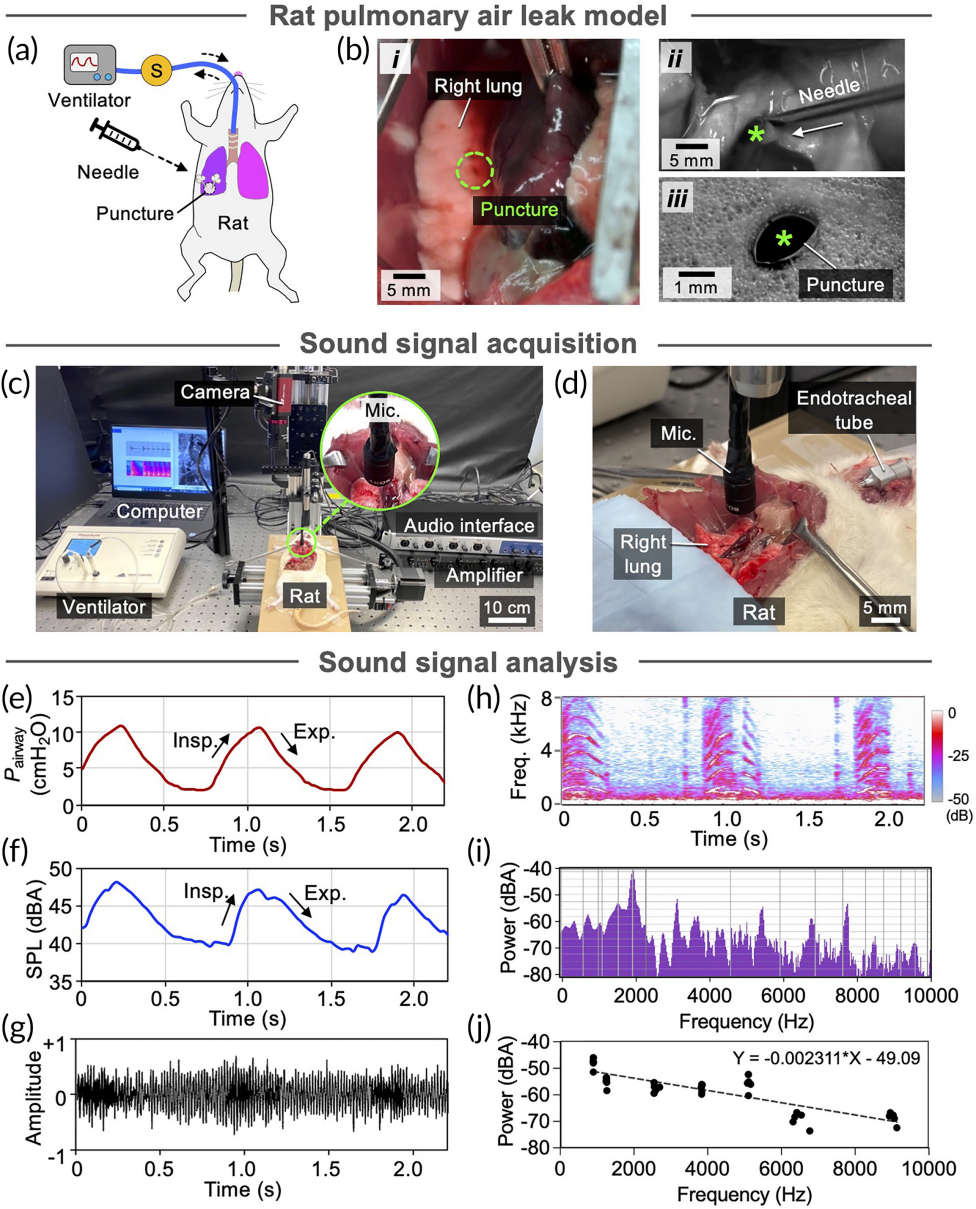
Analysis of pulmonary air leak sounds in rat lungs. (a) Schematic of needle puncture and air leak induction in rat lung. S: pressure and flow sensors. (b) (*i*) Puncture wound in rat lung. (*ii*) Air leak was induced by puncturing the lung with a needle (18‐gauge or 16‐gauge). (*iii*) Focal puncture wound (diameter: 1.3 mm). (c) Photograph of setup to monitor pressure and record air leak sounds in rat lungs. (d) Photograph of microphone positioned above the punctured rat lung for sound recording. Mic: microphone. (e) Pressure of inhaled air (*P*_airway_) measured at the trachea during sound recording. Insp: inspiration. Exp: expiration. (f) A‐weighted sound pressure level (SPL). (g) Normalized amplitude of the acquired sound signal. (h) Spectrogram of the air leak sound calculated from the recorded sound showing sound frequency distribution and density. Freq: frequency. (i) Representative air leak power spectra obtained via Fourier transform. (j) Inverse relationship between band power and frequency in air leak power spectra (*P* < 0.001, *R* ^2^ = 0.702). *Y* = −0.00231*X* – 49.1, where *X* is frequency and *Y* is frequency band power

Post‐surgical air leak is caused by a perforation in the visceral pleura that often occurs along or near a staple line due to failure of the staples or tearing of lung tissue.^4^, ^5^ Defects can occur (a) focally at a single staple or (b) along a series of consecutive staples, and range in size from <1 mm to >1 cm. In this study, we investigated small defects resulting from focal puncture (i.e., <1 mm) in a rat model of pulmonary air leak to assess the sensitivity of our system to detect minor leaks that result from failure of a single staple (Figure 2). Rat lungs provide a suitable model to test sensitivity of our methodology and system because air pressures and tidal volumes of rat lungs are significantly lower than those of human lungs, corresponding to lower pressure drop (∆P) across the lung alveoli and thoracic space, resulting in quieter air leak sounds. By validating efficacy of the sound analysis in rat lungs, we demonstrated high sensitivity and accuracy of our system and methodology. Accordingly, to induce air leak in rat lung, we punctured the visceral pleura of intubated rats that were ventilated with a tidal volume of 6 ml/kg at a ventilation frequency of 70 breaths per minute (bpm) using an 18‐gauge (diameter: 1.27 mm) or 16‐gauge (diameter: 1.65 mm) needle (Figure 2b and Video S1). Airway pressures were continuously monitored using a small animal ventilator, pressure sensors, and custom‐written MATLAB scripts (Figure 2a and Figure S4a). Air leaks were confirmed by observation of decreased air pressure and compliance, as indicated by flattened peaks of the pressure curves. Focal puncture wound induced with 18‐gauge and 16‐gauge needle led to declines in peak inspiratory pressure of 39% (from 16.2 to 9.9 cmH_2_O; Figure S4c,d) and 48%, respectively (from 16.2 to 6.7 cmH_2_O; Figure S4e,f).

### 2.2 Detection of pulmonary air leak in rat using acoustic evaluation of air leak sounds

We investigated whether changes in peak inspiratory pressure resulting from air leak would be accompanied by corresponding changes in sound frequency at the air leak site. Air leak sounds were recorded and analyzed across three respiratory cycles using our custom‐built sound system (Figure 2c, d and Video S3) and all measurements were repeated three times. Because airway pressure (*P*_airway_: 2.1–10.7 cmH_2_O; Figure 2e) was always greater than ambient pressure (*P*_Amb_: 0 cmH_2_O), we presumed that during positive pressure ventilation no air flowed back into the lung through the leak site (Video S2), and that acquired sounds represented the acoustic signal resulting from air escaping through the punctured pleura. Loudness was calculated as sound pressure level (SPL), which was quantified in A‐weighted decibel (dBA), and plotted against time to determine if intensity and periodicity of the sound correlated with *P*_airway_ (Figure 2f). Analysis showed a positive association between *P*_airway_ and SPL as the shapes of these two curves closely resembled each other (Figure 2e,f). We quantified this correlation and determined a positive linear relationship between sound pressure level and air pressure with Pearson correlation coefficient (ρ) of 0.94 (Figure S5). Pressure difference (∆P) between inside (*P*_alveolar_) and outside (*P*_Amb_) the lung is the major driving force that causes air leak. Due to difficulties in accurate measurement of *P*_alveolar_, we instead measured *P*_airway_. While the two values were related, they were not the same. During normal ventilation, there is a pressure drop across the airways, dependent on driving frequency and airway structure. Accordingly, the loudest (47.5 dBA) and quietest (39.8 dBA) acquired sounds corresponded to the maximum (10.7 cmH_2_O) and minimum airway pressure (2.1 cmH_2_O), respectively, in each respiratory cycle. Loudness of the air leak sounds increased more rapidly during inspiration (24.84 dBA/s) than expiration (14.53 dBA/s), which suggested that air leak loudness depended on ventilation parameters (i.e., *P*_airway_, tidal volume, respiratory rate) which contributed to the total volume of air loss through the pleural defect.

To quantify air leak sound signatures, we analyzed the frequency distribution of the air leak sounds acquired from the rat model of air leak through spectral analysis (Figure 2g–j). Sound spectrograms were generated to assess time‐varying intensity and frequency throughout the respiratory cycle. The spectrograms revealed that the air leak sounds contained a group of sound bands, each with a distinct frequency that increased and then decreased in patterns similar to those of *P*_airway_, amplitude, and sound loudness (Figure 2h). This suggested that both loudness and frequency of air leak sounds could be used to characterize and quantify air leak. We then extracted the air leak sound near the plateau of the curves in the spectrogram (Figure 2h, dotted region in Figure S6a) and obtained power spectra to compare relative intensity of the frequency bands (Figure 2i and Figure S6b). We plotted the regions of air leak sound spectrograms that contained frequency bands, which consistently corresponded to inspiratory peaks. Seven distinct frequency bands (fb1–fb7) with amplitude above −80 dB were identified between 0 and 10 kHz, each with narrow and concentrated frequencies. The lowest frequency band (i.e., fb1: 890 Hz) had maximum intensity among the seven bands, and was determined to be noise generated by the ventilator. While intensity of the sound bands generally decreased with frequency, the band that centered at 5020 Hz (fb5) had the second strongest intensity. A harmonic sound consists of a series of sound frequencies at integer multiples of the fundamental frequency.^18^ The ratios of fb3–fb7 (frequency range: 2524–8845 Hz) to fb2 (frequency: 1223 Hz) were approximately 2, 3, 4, 5, and 7, respectively, which indicated that these sound bands were a harmonic series. To determine the relationship between band frequency and sound intensity, we performed a linear regression on the identified frequency bands generated by air leak in rat, and found a statistically significant inverse correlation (Figure 2j, Y = −0.00231X – 49.1, where X is frequency and Y is frequency band intensity, R^2^ = 0.702, *p* < 0.001).

### 2.3 Swine model of pulmonary air leak

**FIGURE 3:**
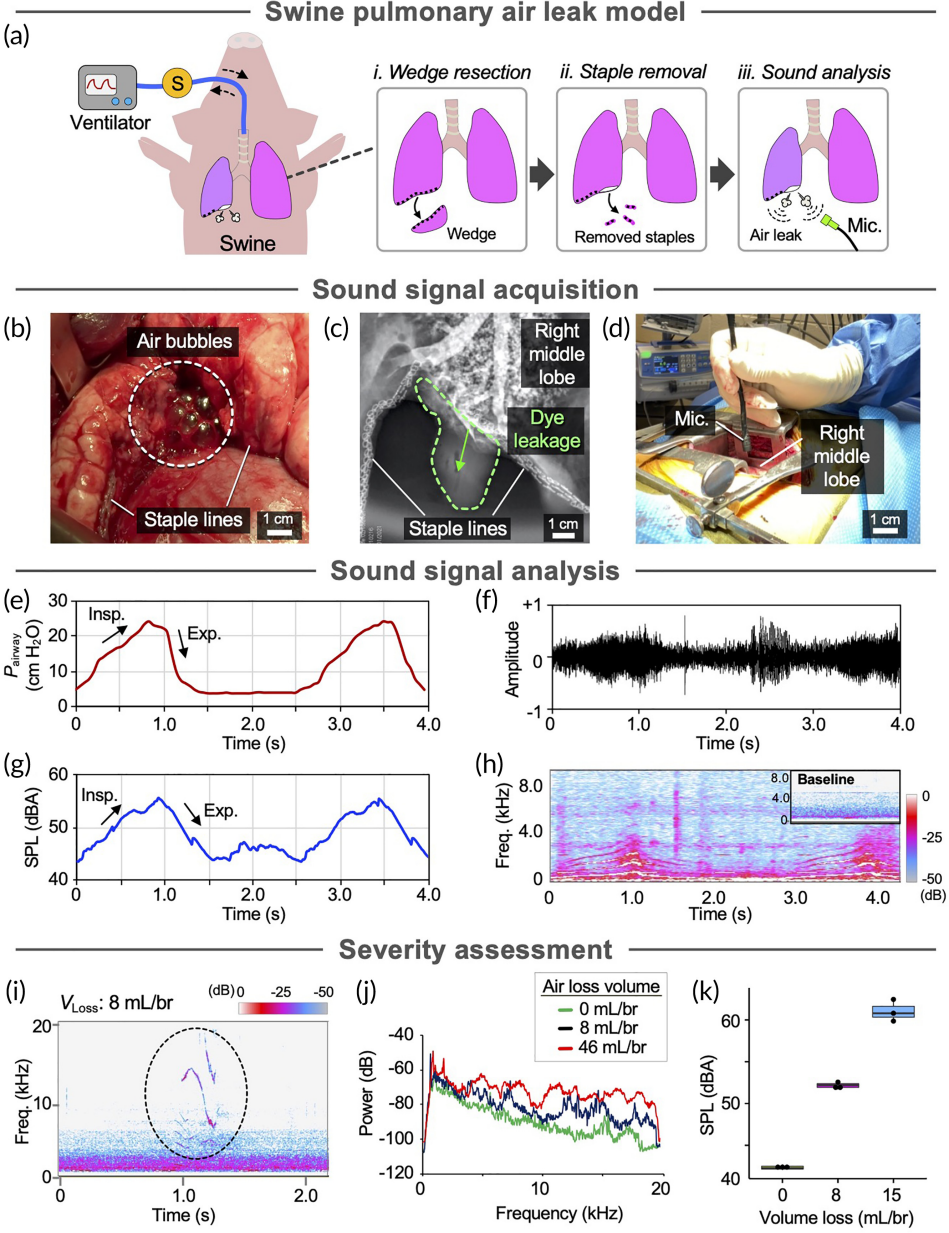
Analysis of pulmonary air leak sounds in swine lung staple line failure model. (a) Schematic of swine wedge resection and staple line failure model to induce pulmonary air leak for sound analysis in situ. S: pressure and flow sensors. (b) Photograph of right middle lobe with air bubbling at site of staple line failure air leak. (c) Radiograph of radiopaque dye leaking from the lung parenchyma at site of staple line failure air leak. (d) Photograph of air leak sound acquisition in situ. Mic: microphone. (e) Pressure of inhaled air (*P*_airway_) measured at the trachea during sound recording. Insp: inspiration. Exp: expiration. (f) Normalized amplitude. (g) A‐weighted sound pressure level (SPL). (h) Spectrogram of the air leak sound calculated from the recorded sound showing sound frequency distribution and density. Freq: frequency. (i) Spectrogram showing sound frequency distribution and density of mild air leak. Freq: frequency. *V*_Loss_: tidal volume loss. Br. breath (j) Power spectra of air leaks of varying severity. (k) loudness of air leak sounds of varying severity

Swine are often used as a model for lung injury because the anatomy, size, and respiratory parameters are similar to those of human lungs.^19^ We assessed if similar air leak sounds occurred in human‐sized lungs and determined that the harmonic characteristic is a distinct, quantifiable feature of air leak sounds using a clinically relevant swine model of pulmonary air leak. The swine model (a) mimiced airway pressures and volumes observed in human lungs, (b) recapitulated post‐surgical air leak due to staple line failure, and (c) occurred in situ (Figure 3a). Wedge resection of the right middle lobe was performed using a surgical stapler (Figure S7a,b). By removing up to 30% of the staples (Figure S7c), we induced staple‐line failure air leak that resulted in substantial decrease in average tidal volumes which was measured using the ventilator (i.e., 45.2 ml/breath or 27.4% loss; *p* < 0.003; Figure S7d). Swine lungs were ventilated with a positive end expiratory pressure (PEEP) of 5 cm H_2_O for all studies. Air leak was confirmed through direct observation of air bubbles at the air leak site (Figure 3b and Video S4) and by radiographic visualization of radiopaque dye leakage following bronchoscopic delivery (Figure 3c).

Detection of pulmonary air leak in swine using acoustic evaluation of air leak sounds. We then assessed air leak sounds in a clinically relevant setting by applying our methodology in human‐sized (i.e., swine) lungs in situ. Following staple line failure, we obtained intensity, loudness, and spectrograms of air leak sounds using our sound analysis system (Figure 3d and Figure S7e). Swine lungs were ventilated at 15 bpm while airway pressure and tidal volume was continuously monitored (Figure 3e, Table S1, and Video S5), and readouts obtained from at least two breathing cycles were extracted and plotted with respect to time. Consistent with observations in rat lungs, amplitude (Figure 3f), calculated loudness level (Figure 3g), and spectrogram (Figure 3h) of the air leak sounds closely correlated with ventilation parameters, as sound intensity (loudness) corresponded with *P*_airway_ during ventilation (Figure 3e). We again determined a positive linear relationship between sound pressure level and airway pressure with Pearson correlation coefficient (ρ) of 0.95 (Figure S8). Loudness of air leak sounds in swine lungs (range: 43.4–55.5 dBA) was greater than that of rat lungs (range: 39.8–47.5 dBA), possibly due to greater ∆P (∆P_swine_ = 20.2 cmH_2_O; ∆P_rat_ = 18.6 cmH_2_O). Swine lung spectrograms showed that air leak sound signature contained approximately four frequency bands between 0 and 5 kHz that fluctuated with *P*_airway_ (Figure 3h). While the frequency bands extended beyond 8 kHz in rat lungs (Figure 2h and Figure S6), the bands predominantly remained below 4 kHz in swine lungs, possibly due to difference in defect size (20 mm in swine vs 1.3 mm in rat), as oscillation frequency by airflow is inversely proportional to defect length.^16^, ^17^ Frequency bands with amplitude above −60 dB identified at the plateau (i.e., fb1–4) were extracted and plotted in a power spectrum (Figure S9a), in which sound intensity generally decreased with frequency, as in rat air leaks (Figure S6). The fundamental frequency band (fb1: 665 Hz) generated the highest intensity sound. The ratios of fb2–4 (frequency range: 1355–2731 Hz) to fb1 were approximately 2, 3, and 4, respectively, comprising a harmonic series (Figure S9b). We performed a linear regression on the identified frequency bands generated by air leak in swine, and found a statistically significant inverse correlation between band frequency and sound intensity (Figure S9c, *Y* = −0.00792*X* – 34.8, where *X* is frequency and *Y* is sound band intensity, *R* ^2^ = 0.896, *p* < 0.001).

### 2.4 Assessment of air leak severity in swine lung using acoustic analysis

Air leak severity is typically described by the volume of air escaping the lung from the leak site.^20^ To assess air leak severity using our sound‐guided method, we modulated volume loss and analyzed the effects on air leak sounds. For a mild leak (air volume loss of 8 ml/breath), we detected multiple frequency bands of reduced sound intensities compared to those detected in severe air leak (Figure 3i,j and Videos S6 and Video S7). Transient high frequency spectral bands were found in the absence of air leak, as normal breath sounds display energy in broad bands depending on air flow, chest wall thickness, and location of sound acquisition (Figure S10a and Video S8). On the other hand, in both mild (volume loss: 8 ml/breath; Figure S10b) and moderate (volume loss: 46 ml/breath; Figure S10c) air leaks, discernible frequency bands were observed on spectral analysis. We also noticed that sounds generated due to bursting of air bubbles near air leak site were visualized as multiple vertical lines in the spectrogram (Figure S10c). Further, we assessed the sensitivity of our sound‐guided method in distinguishing subtle differences of air volume loss. Results showed that our method can accurately differentiate two different levels of air volume loss representative of mild air leak by comparison of sound intensity (52.3 dBA for 8 ml/breath and 62.1 dBA for 15 ml/breath; Figure 3k). Collectively, the results suggest that sound‐guided detection and quantification of pulmonary air leak can enable rapid and accurate assessment and differentiation of air leak severity with a high degree of sensitivity.

### 2.5 Precise localization of pulmonary air leak via sound intensity measurements

**FIGURE 4:**
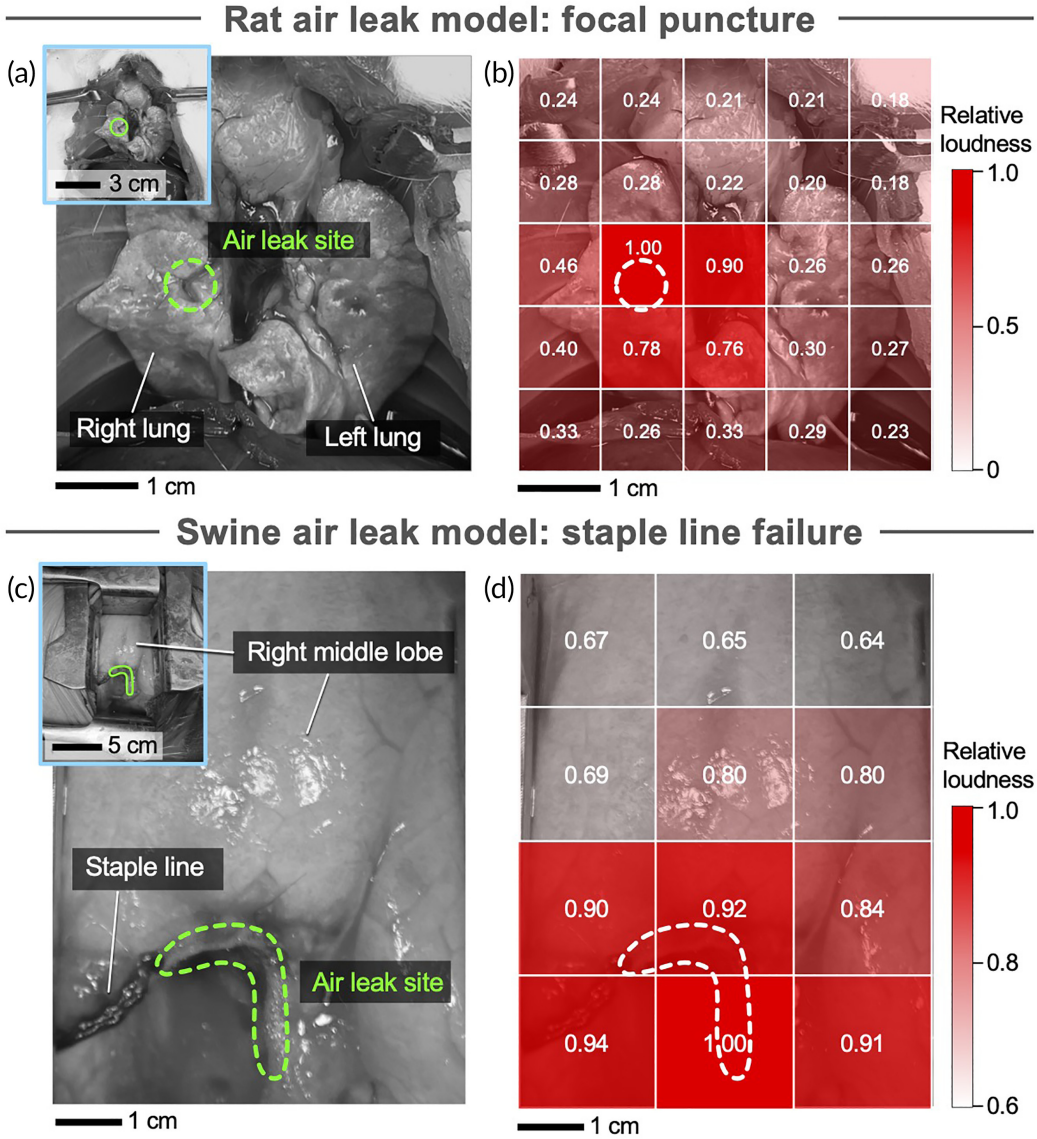
Localization of air leak site by measurement of relative loudness. (a) Rat lung photograph and (b) corresponding breath sound intensity matrix heat map. (c) Swine lung photograph and (d) corresponding breath sound intensity matrix heat map. The value in each array indicates the measured loudness normalized to the maximum loudness measured at the air leak site (dotted regions). Inset: overview photographs of analyzed regions of lung

Clinical methods, such as X‐ray and ultrasound, detect the presence of pneumothorax resulting from air leak, and can only determine if the leak is in the left or right lung. Precise identification of the exact air leak site could facilitate targeted intervention and thus improve patient outcomes. Therefore, we investigated whether quantitative sound analysis could enable high‐resolution localization of air leak site (Figure 4). Rat lungs with focal puncture air leak were continuously ventilated while a breathing sound intensity matrix was constructed. To generate breathing sound intensity matrix, we systematically measured the loudness of air leak sounds across a matrix with step size 1 cm. Starting from the upper left region of the lung, we recorded sound for 5 seconds and calculated average loudness. The microphone was then advanced to an adjacent square (e.g., 1 cm to the right), and sound acquisition and loudness quantification were repeated to obtain a heatmap showing distribution of the normalized sound intensity, ranging between 0 (no sound) and 1.00 (maximum sound), across the whole lung. To establish the baseline breathing sound intensity matrix, analysis was first performed in uninjured ventilated lungs (Figure S11a,b), where all sound intensity scores ranged 0.19–0.20. Sound intensity analysis was repeated in lungs punctured with an 18‐gauge needle. In the breathing sound intensity matrix of lungs with air leak, sound intensity strongly correlated with spatial proximity to the air leak site. Specifically, sound intensity in lungs with air leak ranged 0.18–1.00, where intensity of 1.00 corresponded to the precise location of air leak. This method consistently resulted in precise localization of air leak sites within 1 cm (Figure 4a,b).

We similarly assessed the ability of our system to locate the site of air leak using sound intensity measurement in human‐sized swine lungs. We performed right middle lobe resection and measured sound loudness at 12 points prior to induction of air leak. To demonstrate sensitivity, we performed localization analysis on a mild air leak, which was achieved by removing staples to obtain a 15 ml/breath of air leak. Sound intensity measurements were repeated at the same 12 locations following induction of mild air leak. Normalized sound intensity (range: 0.65–1.00) was greater near the region immediately surrounding the site of air leak (within ~2 cm of leak site; Figure 4c, d), whereas sound intensity (range: 0.37–0.40) did not differ regionally in the lung without air leak injury (Figure S11c,d).

### 2.6 Elimination of heartbeat sound via noise canceling

During air leak sound acquisition and analysis in clinical settings, confounding sound signals originating from background noise (e.g., equipment, other auscultative sounds) can contaminate the air leak sound signals. In particular, while the intensity of background noise can be reduced during measurements, sounds generated by the heart could interfere with measurements due to proximity to the lung. To address this potential issue, we investigated if air leak sound could be detected and isolated in the presence of heart sounds (Figure S12a and Video S9). We generated a sound mixture by adding air leak sound signals collected from swine with staple‐line failure (Figure 3h) and heart sounds obtained from a healthy human (Figure S12b–d). Spectral analysis of the sounds revealed that frequency of human heart sounds remained below 400 Hz. This substantial difference in frequency range between heart sounds and pulmonary air leak sounds allowed us to effectively attenuate the heartbeat noise using high‐pass filtering to extract the air leak signal (Figure S13a–c and Video S10).

## 3 DISCUSSION

Auscultation of heart and lungs, typically with a stethoscope, has been used for cardiopulmonary evaluation and diagnosis for over a century,^21^ but remains reliant on the subjective experience of the care provider.^22^ Leveraging the established principle that respiratory sounds can offer diagnostic insight, we developed an objective, quantitative sound analysis methodology that can discern distinct sound signatures of pulmonary air leaks based on key parameters such as frequency distribution and loudness level (Figure 1). We evaluated air leaks in both small and large animal models to demonstrate the high sensitivity and clinical relevance of this methodology, and determined: (a) air leaks generate discernable sounds that contain harmonic series, (b) air leak sound properties correlate with volume of air loss, and thus leak severity (Figures 2 and 3), and (c) air leak loudness correlates with distance from the leak, enabling in situ identification of leak site (Figure 4). To maximize clinical relevance, we investigated pulmonary air leaks in a swine model due to the anatomical and respiratory (e.g., airway pressure, compliance, tidal volume) similarities to human lungs.^19^ To emulate a realistic hospital setting with noisy environment, all studies were conducted in an operating room with standard medical equipment and background noises.

In pulmonary air leaks, air escapes the lung through a defect in a bronchial staple line or the visceral pleura due to a pressure gradient between the alveoli and thorax. If sufficient kinetic energy is transferred from the airflow to surrounding pleural tissue, the tissue oscillates at its resonance frequency which can generate audible sounds (similar to vocal cord vibration during phonation) (Figure S2). Our results obtained from both small and large lungs consistently showed that sounds generated due to air leak were primarily dependent on the scale of the driving force (i.e., the pressure difference between inside and outside the lungs, ∆P) and the pleural defect geometry (size, shape), which together modulated the resulting sound loudness, pitch, and spectral density (Figures S5–S10). We applied signal processing algorithms, including noise filtering, to isolate air leak sound signatures for analysis, as envisioned clinical use of this methodology would require filtration of ambient sounds from the patient's heart, medical equipment, and other background noise.^21^ This study applied a high‐pass filter (cutoff frequency, f_c_ = 500 Hz) to attenuate recorded heart sounds for analysis of air leak in an open thoracotomy. By tuning algorithm parameters such as attenuation rate and cutoff frequency of the filter, we detected distinct air leak sound frequency patterns and spectral density profiles, and confirmed that air leak sounds could be isolated from a sound mixture using a high‐pass filtering algorithm (Figures S12 and S13). Additional advanced noise cancellation methods, such as active or adaptive noise canceling, could be implemented to improve signal‐to‐noise ratio. While band pass filters and spectral analysis have previously been applied to process heart and lung sounds, our methodology is the first designed to quantitate and characterize pulmonary air leak sounds.^23^, ^24^, ^25^, ^26^, ^27^

Notably, in both rat and swine models, we discovered that air leak sounds contained harmonic series, in which each frequency was an integer multiple of the fundamental frequency of the oscillating pleural tissue (Figures S6 and S9).^18^ To the best of our knowledge, this is the first report of harmonic series as a feature of pulmonary air leak. We also determined a statistically significant inverse correlation between sound power and band frequency in both rat and swine (Figure 2j, Figure 3j,k, and Figure S9c). The correlation between frequency and relative intensity of bands in air leak harmonic series, as well as the absolute value of the fundamental frequency, may correspond to physical and pathological features, such as leak location, geometry, underlying lung disease, and tissue stiffness. The fundamental frequency and relative intensity of bands in these harmonic series of air leak sounds may correspond to physical and pathological features, such as leak location, geometry, underlying lung disease, and tissue stiffness. Although the mechanisms of air leak resolution remain to be elucidated, healing likely depends on anatomical factors, as mechanical strain and ventilation are not uniformly distributed throughout the lung,^28^, ^29^ and also on patient‐specific factors such as age, underlying lung disease, co‐morbidities, immunocompetency, and need for positive‐pressure ventilation. Air leak sound signatures and harmonics may correlate with these variables and be used to better understand and predict air leak healing in an individualized manner.

Prior to beginning this study, we interviewed 66 practicing healthcare providers who treat patients with pulmonary air leak (e.g., surgeons, pulmonologists, emergency medicine, nurses, etc.) to confirm the clinical need for improved methods of detecting, assessing, and treating pulmonary air leak (Figure S14). This study was therefore motivated by the fact that existing modalities for detecting air leaks did not allow for precise assessment or localization, and established air leak scoring systems, which were based on nominal categories and analog graduations, were not widely used due to high inter‐observer variability^30^, ^31^, ^32^ (Tables S2 and S3). Methods for intraoperative assessment of air leaks rely on submerging the lung in water or exogenous surfactant (i.e., Yang's bubble solution) and visualizing air bubbles that form at the leak site. Results of these air leak tests can be difficult to assess, particularly when direct visualization is difficult, including in peripheral or posterior lung regions or during thoracoscopic [video‐assisted thoracoscopic surgery (VATS)] procedures.^6^, ^33^ Additionally, these methods are qualitative and subjective (Table S4). Digital chest tube drainage systems attempt to quantitate air leaks, however, are limited by high cost and variable results. No current modality enables precise localization of air leak sites. Diagnostic modalities such as auscultation, X‐ray, computed tomography (CT) scan, and ultrasound can only detect the presence of pneumothorax, and offer minimal or no information about air leak location or severity—a gap in diagnostic capabilities that significantly limits air leak treatment (Table S5).^6^, ^34^, ^35^, ^36^, ^37^, ^38^, ^39^, ^40^ Here we demonstrate precise localization of air leak site within 1 cm. Finer resolution may be achievable with a smaller microphone or decreased step size in sound intensity matrix measurements. Existing adjunct interventions (e.g., applied suction, pleurodesis, polymer sealant) are inconsistently used due to the lack of objective metrics for leak assessment, difficulty predicting which patients may benefit, and inability to target delivered treatments to the injured region. As a result, clinical management of air leak is often conservative, relying on continued chest tube drainage of air and fluid until the leak heals on its own.^41^, ^42^ In comparison, the proposed sound‐guided analysis would offer an objective, quantitative methodology for assessing air leak severity and location which can aid in development of air leak management strategies, enable targeted delivery of adjunct therapeutics, and predict which patients may require additional interventions such as pleurodesis or re‐operation.

While findings of this proof‐of‐concept study demonstrate potential for eventual translation, we acknowledge several limitations and identify future research directions to further strengthen clinical utility of this methodology. Air leaks were only evaluated in the right lung of healthy swine during positive‐pressure ventilation. Future studies should assess how air leak sounds differ across lung regions and pathologies, and determine if similar air leak sound signals can be detected in lungs during normal ventilation without mechanical support. Effects of different ventilatory conditions (e.g., peak inspiratory pressure, positive end‐expiratory pressure, tidal volumes) on air leak sound intensity and frequency distribution can be investigated to provide comprehensive explanation of the correlation between ventilation regimes and acoustic signatures of air leak. During sound recording from swine lungs, microphone positions were manually manipulated and maintained 1 cm from the surface of the lung. Because measured sound intensity can be influenced by the location of the microphone with respect to measurement site (e.g., distance between microphone and lung surface), establishment of optimized algorithms, standard operating protocols, and precise upper and lower sound detection limits could further reduce errors and variabilities between measurements.^43^ Detection limits of our methodology would be influenced by aerodynamic interactions between the air and the surrounding tissue at the leak site, sensitivity of the sound acquisition instruments (e.g., microphone, signal amplifier), and the quality of the recordings and noise cancelation methods. Lastly, because all air leak sounds were investigated intraoperatively during non‐survival studies with open thoracotomy, changes in air leak sound signatures over extended periods of time and transthoracic air leak sounds were not evaluated. Longitudinal studies can further elucidate correlations between air leak sound properties and post‐operative healing within the closed chest.

Based on our finding that air leaks generate unique, quantifiable sound signatures, we envision that this sound analysis methodology could be adapted for use in: (a) open chest surgery, (b) minimally invasive surgery (VATS), or (c) non‐invasively through closed chest. The application that we explicitly demonstrate in this study is the use of our device in (a) open chest surgery (i.e., thoracotomy; Table S6). While the water submersion test is used as the standard method for intraoperative assessment of pulmonary air leak, it can be challenging to visualize peripheral or posterior lung regions, and many air leaks may be missed. Our system overcomes this challenge because it can detect air flow from the leak without visualization of the leak site. Our method could be easily adapted for use in (b) VATS procedures. Standard thoracoscopic ports were 5–12 mm, and the microphone used in this study was 5 mm in diameter. Thus, a smaller microphone may be required to accommodate all surgical instruments that need to pass through the port. The application in VATS is particularly compelling as methods for detecting pulmonary air leak intraoperatively, such as the water submersion test or Yang's bubble solution test, are challenging to perform during VATS procedures. For lung cancer resection in the United States alone, an estimated 66,000 patients undergo open lung surgery each year and an additional 54,000 undergo VATS procedures.^44^ Therefore, a significant patient population could benefit from these applications.

The ability to apply quantitative sound analysis in a non‐invasive manner through a closed chest presents the opportunity to benefit the greatest number of patients. Globally, 20 million patients receive positive pressure mechanical ventilation each year, and are at increased risk for developing pulmonary air leaks due to barotrauma and underlying lung disease.^45^ As pulmonary air leak can rapidly progress to a potentially fatal tension pneumothorax, rapid detection of air leak is imperative (Figure S1). We envision a modified configuration of our sound analysis system in which wearable sensors are placed on the chest to continuously monitor pulmonary sounds to rapidly detect pulmonary air leak. We recognize several challenges toward application of our system in a closed chest due to transmission characteristics of the intervening chest wall, including differential coupling, sound attenuation, and spectral distortion. To address these challenges and enable accurate, non‐invasive analysis of air leak sounds, we propose incorporation of: (a) accelerometer contact microphone array on the chest, (b) high pass filter and adaptive filtering, and (c) triangulation for accurate localization (Figure S15). While the magnitude of the sound signal would be lower in the closed chest, prior work has demonstrated the utility of contact accelerometers for precise, non‐invasive analysis of cardiopulmonary sounds of low magnitudes, and could be extended to use in our system.^46^, ^47^, ^48^ Whereas air coupled microphones are highly sensitive to environmental noises, the incorporation of multiple accelerometers onto the chest wall could enable the use of signal processing techniques including decomposition, independent components analysis, and modulation filtering to allow for precise analysis of sound signals originating from air leak.

## 4 CONCLUSIONS

The goal of this proof‐of‐concept study was to demonstrate that pulmonary air leaks generate unique, quantifiable sound signals. The ability of this method to precisely assess and localize pulmonary air leaks through non‐invasive acoustic signal analysis through the chest wall remains to be studied. Future device iterations will focus on modifying this system and methodology for use in a closed chest. The ability to precisely locate air leaks in a real‐time and minimally invasive manner could enable locally targeted and patient‐specific administration of therapeutics, such as a polymer‐based lung sealant to expedite air leak treatment and improve outcomes. Similar sound detection and analysis methodologies could also be developed for diagnostic evaluations of other organs, such as heart and intestine, where sounds can be evaluated to quantify injury or disease states.

## 5 MATERIALS AND METHODS

Detailed methods can be found in Supporting Information. Briefly, the sound acquisition and analysis system comprised a miniature microphone (diameter: 5.6 mm; length: 3 mm), motorized stage to position the microphone, signal amplifier, audio interface, and custom‐written MATLAB codes (Figure 1a and Figure S3a,b). Notably, standard thoracoscopic ports had inner diameters up to 12 mm, which could allow insertion of the mini‐microphone for minimally invasive sound analysis in situ. Sound signals obtained with the microphone passed through the amplifier and audio interface to the computer for sound processing and quantification using custom‐written MATLAB scripts, which removed ambient noise, amplified signals, and quantified acquired sounds to predict severity and determine air leak location (Figure 1b). To validate this methodology, we applied quantitative sound analysis in rat and swine models of pulmonary air leak and determined specific acoustic signatures associated with air leaks under different injury states and ventilatory conditions. A rat model of pulmonary air leak in open thoracotomy was established by focal puncture with 16‐gauge and 18‐gauge needle. Swine model of pulmonary air leak in open thoracotomy was established by wedge resection of right middle lobe with EndoGIA surgical stapler and 60 mm purple load staples, followed by removal of up to 30% of fired staples to induce air leak. Sound signal originating from rat and swine pulmonary air leaks were recorded with Sony ECM 77B and analyzed using custom‐written MATLAB codes. All animal procedures were approved by and conducted in accordance with the Institutional Animal Care and Use Committee of Stevens Institute of Technology and Columbia University.

## CONFLICT OF INTEREST

No competing interests to disclose.

## AUTHOR CONTRIBUTIONS

**Meghan R. Pinezich:** Conceptualization (equal); data curation (equal); formal analysis (equal); investigation (equal); methodology (equal); validation (equal); visualization (equal); writing – original draft (equal); writing – review and editing (equal). **Seyed Mohammad Mir:** Data curation (equal); investigation (equal); validation (equal); visualization (equal); writing – original draft (equal); writing – review and editing (equal). **Jonathan A. Reimer:** Data curation (equal); investigation (equal); validation (equal); visualization (equal). **Sarah R. Kaslow:** Data curation (equal); investigation (equal); validation (equal); visualization (equal). **Jiawen Chen:** Data curation (equal); formal analysis (equal); validation (equal); visualization (equal). **Brandon A. Guenthart:** Conceptualization (equal); methodology (equal); writing – original draft (equal); writing – review and editing (equal). **Matthew Bacchetta:** Investigation (equal); methodology (equal); writing – original draft (equal). **John D. O'Neill:** Conceptualization (equal); formal analysis (equal); investigation (equal); methodology (equal); validation (equal); visualization (equal); writing – original draft (equal); writing – review and editing (equal). **Gordona Vunjak Novakovic:** Conceptualization (equal); funding acquisition (equal); investigation (equal); methodology (equal); project administration (equal); resources (equal); supervision (equal); validation (equal); writing – original draft (equal); writing – review and editing (equal). **Jinho Kim:** Conceptualization (equal); data curation (equal); formal analysis (equal); funding acquisition (equal); investigation (equal); methodology (equal); project administration (equal); resources (equal); supervision (equal); validation (equal); visualization (equal); writing – original draft (equal); writing – review and editing (equal).

### PEER REVIEW

The peer review history for this article is available at https://publons.com/publon/10.1002/btm2.10322.

## Supporting information


**Figure S1** Progression of pulmonary air leak to pneumothorax and tension pneumothorax. (A) Pulmonary air leak is a common complication of lung surgery and occurs when air leaks from the airspace in the lung into the pleural space. (B) Pneumothorax occurs when intrapleural pressure increases causing lung to collapse. (C) Tension pneumothorax occurs when intrapleural pressure increases significantly, causing the mediastinum to shift and impairing venous return. Tension pneumothorax can cause hemodynamic instability, obstructive shock, and death.
**Figure S2** Generation of air leak sounds. Air leak sounds are generated as air escapes through a defect in the visceral pleura of the lung. Kinetic energy is transferred from the escaping air to the surrounding tissue, vibrating the tissue at distinct frequencies. When airflow‐induced tissue vibration produces sounds that exceed sound pressure ~20 μPa, audible sounds are generated.
**Figure S3** Setup of pulmonary air leak sound analysis system. (A) Schematic of computer‐assisted sound analysis system. DAQ: data acquisition system. S: pressure and flow sensors. (B) Photograph of pulmonary air leak sound analysis system. Inset: Microphone used for air leak sound acquisition.
**Figure S4** Rat pulmonary air leak model. (A) Photograph of setup used to monitor air pressure in rat lungs. (B) Photograph of air leak in ventilated rat lungs. (C) Pressure inside the lung airway (*P*_Airway_) was measured while a focal puncture injury was induced using 18‐gauge (18G) needle. Insp: inspiration. Exp: expiration. (D) Air pressure curves obtained from control rat lung and lung punctured with 18G needle. (E) Pressure curve obtained from lungs injured using a 16‐gauge (16G) needle. (F) Air pressure curves obtained 1 second after injury with 16G needle and 15 seconds post‐injury.
**Figure S5** Evaluation of the correlation between airway pressure and sound pressure level in rat lungs with air leak. (A) Normalized magnitudes (range between 0 and 1) of airway pressure (*P*_*airway*_) and A‐weighted sound pressure level (SPL) displayed in a single plot. (B) Scatter plot showing correlation between normalized *P*_*airway*_ and SPL where the relation is roughly linear as the dots are distributed along a straight line. Pearson correlation coefficient (ρ) was determined to be 0.94, suggesting a strong positive correlation between these two data sets.
**Figure S6** Spectral analysis of pulmonary air leak sounds in rat model. (A) Spectrogram of air leak sound during one breathing cycle with discernible frequency bands (fb1–fb7). (B) Power spectrum of the signal within the dotted region in (A), obtained using Fourier transform. **Figure S7** Swine pulmonary air leak model. (A) Photograph of wedge resection performed on right middle lobe of swine lung using surgical stapler. (B) Photograph of representative resected lung wedge. (C) Photograph of staples (~30% of total) removed from the staple line to induce pulmonary air leak. (D) Change in tidal volume due to pulmonary air leak (n = 4 swine). Mean loss in tidal volume: 27.4%. * *P* = 0.003. (E) Application of sound analysis system to intraoperatively assess pulmonary air leak.
**Figure S8** Evaluation of the correlation between the airway pressure and sound pressure level in swine lung with air leak. (A) Normalized magnitudes (range between 0 and 1) of airway pressure (*P*_airway_) and A‐weighted sound pressure level (SPL). (B) Scatter plot showing linear correlation between normalized *P*_airway_ and SPL. Pearson correlation coefficient (ρ) was determined to be 0.95, suggesting a large positive correlation.
**Figure S9** Spectral analysis of pulmonary air leak sounds in swine model. (A) Spectrogram of the air leak sound during one breathing cycle with discernible frequency bands (fb1–fb4). (B) Power spectrum of the signal within the dotted region in (a), obtained using Fourier transform. (C) Relationship between band frequency and sound intensity (*p* < 0.001). Y = −0.00792X – 34.77, where X is frequency and Y is sound band intensity.
**Figure S10** Spectral analysis of pulmonary air leak sounds in swine model. (A) Spectrogram of baseline with no air leak and thus no tidal volume loss (*TV*_Loss_: 0 ml/breath). (B) Spectrogram of mild air leak with tidal volume loss of 8 ml/breath. (C) Spectrogram of moderate air leak with tidal volume loss of 46 ml/breath. *TV*_Loss_: tidal volume loss.
**Figure S11** Measurement of relative loudness in control lungs with no air leak. (A) Photograph of control rat lung with no air leak and (B) corresponding sound intensity heat map. (C) Photograph of control swine lung right middle lobe with no air leak and (D) corresponding sound intensity heat map.
**Figure S12** Spectral analysis of mixed air leak and heart sounds. (A) Generation of mixed sound by mixing recorded human heart sounds and swine air leak sounds. (B) Spectrogram of heart sounds recorded in a healthy human subject. S1: sound 1. S2: sound 2. (C) Spectrogram of mixed air leak and heart sounds. (D) Amplitude of mixed air leak and heart sounds.
**Figure S13** Extraction of air leak sounds from a mixed sound. (A) Histogram of power spectral density of the mixed sound (air leak and heart sounds: blue) and filtered sound (pink). Filtering of heart sounds was achieved via high‐pass filtering. (B) Spectrogram of the filtered sound. (C) Filtered amplitude to obtain isolated air leak sound.
**Figure S14** Results from survey of healthcare providers (n = 66) treating patients with pulmonary air leak. Survey conducted from June 5, 2020 to July 15, 2020. Survey population included physicians (n = 47), nurses (n = 17), nurse practitioner (n = 1), and physician's assistant (n = 1) across thoracic surgery, general surgery, emergency medicine, and critical care.
**Figure S15** Schematic of envisioned non‐invasive transthoracic air leak sound analysis system. Patient with pulmonary air leak wearing transthoracic accelerometer contact microphone array. Acoustic signals are collected by accelerometer contact microphone sensors, amplified by acoustic preamplifier, and conveyed by audio interface to a computer for acoustic signal processing including high pass and adaptive filtering for quantitative assessment and localization of air leak.
**Table S1** Ventilatory parameters during air leak sound analysis in swine
**Table S2** Robert David Cerfolio (RDC) classification system for air leaks with possible causes, severity, and prevalence of each type of air leak^17^, ^18^, ^19^
**Table S3** Clinical modalities for detection of air leak^20^, ^21^, ^22^, ^23^, ^24^, ^25^
**Table S4** Clinical modalities for intraoperative detection of air leak^26^, ^27^, ^28^
**Table S5** Clinical modalities for detection of pneumothorax^29^, ^30^, ^31^, ^32^, ^33^, ^34^, ^35^, ^36^
**Table S6** Envisioned clinical applications for quantitative auscultation^37^, ^38^

**Video S1** Induction of pulmonary air leak in rat through focal puncture with 18‐gauge needle

**Video S2** Rat model of pulmonary air leak

**Video S3** Air leak sounds from rat model of pulmonary air leak

**Video S4** Swine model of pulmonary air leak. Lungs with air leak were submerged in saline to visualize resultant air bubbles

**Video S5** Air leak sounds from swine model of pulmonary air leak

**Video S6** Air leak sounds recorded in swine with mild pulmonary air leak

**Video S7** Air leak sounds recorded in swine with severe pulmonary air leak

**Video S8** Sounds recorded in swine with normal lungs (i.e., no air leak)

**Video S9** Mixed healthy human heart and swine pulmonary air leak sounds

**Video S10** Air leak sounds filtered from mixed human heart and swine pulmonary air leak sounds

## Data Availability

The authors declare that all data supporting the findings of this study are available within the text, figures, and Supplementary Information. Further, all datasets are deposited in Zenodo (https://zenodo.org/record/5219062#.YR6BKtNJF4s) with doi 10.5281/zenodo.5219062. Access to the deposited datasets will be provided to the editor and reviewers when requested. The datasets will be openly accessible to the public on acceptance of the manuscript for publication.
