# Supplementary material for: Sound‐guided assessment and localization of pulmonary air leak
Source: Bioeng Transl Med. 2022 May 4;8(1):e10322. doi: 10.1002/btm2.10322 (PMC9842055; doi:10.1002/btm2.10322)
Supplement: Supplementary file 1 — Figure S1 Progression of pulmonary air leak to pneumothorax and tension pneumothorax. (A) Pulmonary air leak is a common complication of lung surgery and occurs when air leaks from the airspace in the lung into the pleural space. (B) Pneumothorax occurs when intrapleural pressure increases causing lung to collapse. (C) Tension pneumothorax occurs when intrapleural pressure increases significantly, causing the mediastinum to shift and impairing venous return. Tension pneumothorax can cause hemodynamic instability, obstructive shock, and death. Figure S2 Generation of air leak sounds. Air leak sounds are generated as air escapes through a defect in the visceral pleura of the lung. Kinetic energy is transferred from the escaping air to the surrounding tissue, vibrating the tissue at distinct frequencies. When airflow‐induced tissue vibration produces sounds that exceed sound pressure ~20 μPa, audible sounds are generated. Figure S3 Setup of pulmonary air leak sound analysis system. (A) Schematic of computer‐assisted sound analysis system. DAQ: data acquisition system. S: pressure and flow sensors. (B) Photograph of pulmonary air leak sound analysis system. Inset: Microphone used for air leak sound acquisition. Figure S4 Rat pulmonary air leak model. (A) Photograph of setup used to monitor air pressure in rat lungs. (B) Photograph of air leak in ventilated rat lungs. (C) Pressure inside the lung airway (P Airway) was measured while a focal puncture injury was induced using 18‐gauge (18G) needle. Insp: inspiration. Exp: expiration. (D) Air pressure curves obtained from control rat lung and lung punctured with 18G needle. (E) Pressure curve obtained from lungs injured using a 16‐gauge (16G) needle. (F) Air pressure curves obtained 1 second after injury with 16G needle and 15 seconds post‐injury. Figure S5 Evaluation of the correlation between airway pressure and sound pressure level in rat lungs with air leak. (A) Normalized magnitudes (range between 0 and 1) of airway pr [file BTM2-8-e10322-s002.docx]

**Supporting Information**

**Sound-guided assessment and localization of pulmonary air leak**

Meghan R. Pinezich^1,8^, Seyed Mohammad Mir^2,8^, Jonathan A. Reimer^1,3^, Sarah R. Kaslow^1,3^, Jiawen Chen^2^, Brandon A. Guenthart^4^, Matthew Bacchetta^5^, John D. O’Neill^6^,

Gordana Vunjak-Novakovic^1,7^, and Jinho Kim^2^*

^1^Department of Biomedical Engineering, Columbia University, New York, NY, USA. ^2^Department of Biomedical Engineering, Stevens Institute of Technology, Hoboken, NJ, USA. ^3^Department of Surgery, Columbia University Medical Center, New York, NY, USA. ^4^Department of Cardiothoracic Surgery, Stanford University, Stanford, CA, USA. ^5^Department of Thoracic Surgery, Vanderbilt University, Nashville, TN, USA. ^6^Department of Cell Biology, State University of New York Downstate Medical Center, Brooklyn, NY, USA. ^7^Department of Medicine, Columbia University Medical Center, New York, NY, USA.

^8^These authors contributed equally: Meghan R. Pinezich, Seyed Mohammad Mir.

***Corresponding author:** Jinho Kim, PhD

**Address:** 1 Castle Point Terrace, Hoboken, NJ 07030

**Phone:** 1-201-216-5518

**Email:**  jkim6@stevens.edu

**Content**

**I. Supplementary Discussion**

**II. Supplementary Materials and Methods**

III. Supplementary Figures and Tables

IV. Supplementary Video Captions

**V. Supplementary References**

**I. Supplementary Discussion**

Challenges remain towards individualized management of air leaks with adjunct interventions such as applied suction and pleurodesis. A common problem with air leak patients supported with positive pressure ventilation is optimizing chest tube drainage using suction. While suction can evacuate air to effectively prevent pneumothorax, suction can also negatively impact minute ventilation by creating a positive pressure inflow on a ventilator that is immediately suctioned through a negative pressure chest tube. This prevents the patient from ventilating their lungs, which can lead to hypoxemia, hypercapnia, atelectasis, and consolidation. Due to difficulties in accurate assessment of leak severity, challenges remain toward optimizing suction protocols catered to individual patients.

To accelerate air leak resolution, some providers will perform pleurodesis, in which a second injury is induced on the inside of the chest wall such that scar tissue forms to adhere the lung pleura to the inside of the chest wall to seal the leak and prevent lung collapse. However, difficulties assessing air leak severity make it challenging to determine which patients would require pleurodesis and which patients may have air leaks that will resolve without further intervention. Due to the lack of objective criteria, the decision to administer pleurodesis is often based on physician or institutional preference. Some patients who receive pleurodesis may have air leaks that would otherwise resolve on their own. For these patients, the induction of a second tissue injury may be unnecessary.

Patients who develop air leak often have underlying lung disease, such as chronic obstructive pulmonary disease (COPD), interstitial lung disease (ILD), cystic fibrosis (CF), or acute respiratory distress syndrome (ARDS) secondary to lung injury including COVID-19. Depending on underlying pathology, ventilation strategy can differ dramatically among patients. For example, management of COVID-19 and ARDS patients relies on ventilation with low tidal volume^1^. In order to extract meaningful information from air leak sound signatures, standardized ventilation and measurement protocols must be developed. For example, the impact of positive end expiratory pressure (PEEP), respiratory rate, and ventilatory mode should be considered. Further investigation is needed to optimize ventilatory management for sound analysis across patient populations.

Various pathologies can contribute to changes in lung tissue stiffness (e.g., emphysema, pulmonary fibrosis)^2^. Tissue stiffness likely impacts sound signatures because stiffer tissue will oscillate differently than more compliant tissue. Additionally, patients with different pathologies may be more susceptible to certain tissue defect geometries that lead to different air leak ‘densities’. COPD patients may be prone to diffuse air leaks that are distributed evenly across long staple lines, whereas ILD patients may have more focal air leaks at the site of an individual staple. Correlating underlying pathology, tissue stiffness, and air leak sound profile could enable precision diagnosis in the future.

**II. Supplementary Materials and Methods**

**Rat model of pulmonary air leak.** Sprague-Dawley rats (*n* = 6, equal numbers of male and female animals, 200–250 g; SAS SD Rats, Charles River Laboratories) were used for the rat model of pulmonary air leak. All procedures were performed in accordance with the animal welfare guidelines and regulations of the Institute for Animal Care and Use Committee (IACUC) at Stevens Institute of Technology and Columbia University. Rats were euthanized with 5% isoflurane (VED1350, Penn Veterinary Supply) for 30 minutes using a vaporizer (SomnoSuite, Kent Scientific). An endotracheal cannula (73-2727, Harvard Apparatus) was inserted into the trachea and secured using a 6-0 polypropylene suture. Rats were ventilated using a small animal ventilator (PhysioSuite, Kent Scientific; or Inspira, Harvard Apparatus) with tidal volume of 6 mL/kg at a rate of 70 breaths per minute (bpm). A sternotomy was performed using surgical scalpel and scissors to open the chest. Pulmonary air leak was induced by focal puncture of the visceral pleura of the lung (penetration depth: ~ 3 mm) using an 18-gauge (diameter: 1.27 mm) or 16-gauge (diameter: 1.65 mm) needle. Air leak was confirmed by submerging the lung in phosphate-buffered saline (PBS) solution and directly visualizing air bubbles generated at the needle insertion site. Air leak sounds were recorded using our custom-built sound recording and analysis system. All experiments were repeated at least three times.

**Rat lung pressure-volume measurements.** To monitor the pressure-volume (*P-V*) relation of the rat lungs, a sensing module was constructed and connected to the ventilator and endotracheal cannula via a three-way stopcock (BD). The module consisted of a pressure sensor (pressure range: ±7 kPa; MPXV7007GC6U, NXP), airflow sensor (flow range: ±750 cm^3^/min, HAFBLF0200CAAX5, Honeywell), digital acquisition device (DAQ, Arduino Uno), and a computer (XPS 8940, Dell). Pressure and volume data were transferred to the computer via DAQ, then processed and plotted using custom-written MATLAB code (MathWorks). Prior to the measurements, pressure and flow sensors were calibrated according to manufacturer protocol.

**Swine model of pulmonary air leak.** Yorkshire swine (*n* = 4, female, 35–45 kg, Animal Biotech Industries) were sedated with Telazol (5 mg/kg), anesthetized with 5% isoflurane, intubated with endotracheal tube (7.5 mm), and placed in the left lateral position to access the right lung. Swine lungs were ventilated with a positive end expiratory pressure (PEEP) of 5 cm H_2_O for all studies. Thoracotomy was performed in sterile fashion with a curvilinear incision along the fifth intercostal space. Subcutaneous tissue, muscle, and parietal pleura were dissected, and a rib spreader was used to expose the right middle lobe. A wedge resection of the right middle lobe was performed using a surgical stapler with 60 mm (purple) staple loads (Endo GIA^TM^, Covidien) to remove ~15 g of lung tissue. To induce air leak that mimics staple-line failure, up to 30% of the staples were removed using forceps and scalpel until tidal volume decreased from baseline by at least 25%. To confirm presence of air leak, the right middle lobe was submerged in normal saline to visualize bubbles. Radiopaque dye (Omnipaque, GE) was delivered using 3.8-mm flexible video bronchoscope (aScope 3; Ambu) to the right lingular bronchus and dye leakage was visualized using portable X-ray unit (PXP-16HF; United Radiology Systems) at 2.2 mAs and 90 kVp. Air leak sounds were recorded *in situ* with the sound analysis system. All measurements were repeated at least three times. The study received approval from the Institutional Animal Care and Use Committee (IACUC) at Columbia University. All animal care and procedures were conducted in accordance with the US National Research Council’s Guide for the Care and Use of Laboratory Animals, 8th edn.

**Air leak sound recording system and signal acquisition.** A sound measurement platform was constructed by integrating a sound recorder/analyzer network and motorized manipulator. The sound recorder/analyzer consisted of a microphone (Sony ECM-77B, Sony), preamplifier (B12A, Black Lion Audio), audio interface (Fireface 802, RME), and computer (XPS 8940, Dell). Sound signals acquired via the microphone were fed into the preamplifier input channel with maximum gain of 70 dB. Amplified signals were supplied to the audio interface that was connected to the computer via USB cable. During sound measurements in rat lungs, the microphone was mounted on a motorized XYZ manipulator (MiniMill, OpenBuilds) for positioning of the microphone with respect to the measurement site (i.e., air leak site). The motorized manipulator positioned the microphone with submillimeter resolution in three-dimensional space using three integrated motors (total 3 motors; Nema 23 Stepper motor) controlled via Machine Interface Controller software (OpenBuilds). During sound measurements in swine lungs, a human operator positioned the microphone manually. The distance between the microphone tip and operator hand was maintained at 5 cm for all measurements to minimize variation. To maximize signal acquisition and minimize ambient noise, the microphone was positioned perpendicular to the pleural surface 1 cm from the lung pleural surface for all measurements in rat and swine. Audio signals acquired from intact lungs (control) or lungs with air leak were processed for sound analysis and quantification in real time or stored in WAV file format for subsequent sound analysis using custom-written MATLAB codes or open-source audio analysis software (Audacity or Sonic Visualizer). For accurate signal quantification, sound signals were acquired for 5 or 30 seconds (depending on the experiment) through one audio channel with 44.1-kHz sampling rate and 16-bit resolution.

**Analysis and visualization of sound spectrogram.** To investigate acoustic characteristics of the acquired air leak sounds, spectrograms of the sounds were obtained by extracting and plotting amplitude and frequency distributions of the recorded sound signals against time. Frequency content of the sound waveform that varied with time was computed via the Short-Term Fourier Transform (STFT) of the input audio signals^3,4^. Briefly, STFT can be expressed as:

$\text{X}\left[ \text{n}\text{,}\text{ }\text{k} \right]\text{ }\text{=}\text{ }\sum_{\text{m}\text{=0}}^{\text{L-1}} \text{x}\left[ \text{m} \right]\text{w}\text{[}\text{n}\text{-}\text{m}\text{]}\text{e}^{\text{-}\text{j}\text{2}\text{π}\frac{\text{mk}}{\text{N}}}$ [1]

where *x*[*m*] is input signal at time m, L is the length of window function *w*[n*-m*], N is the length of the Discrete Fourier Transform (DFT), and X[*n*, *k*] is DFT of windowed data at reference time *n* and frequency *k* with an integer *N*. Distribution of frequencies contained in the audio signal was visualized using MATLAB or Audacity in the form of spectrograph which is an intensity plot of STFT magnitude over time and can be obtained by |X[*n*, *k*]|^2^. Further, ambient sounds contained in audio signals were removed through filtering^5,6^. For example, to eliminate low-frequency noise, high-pass filter was applied to attenuate sound signals below a cutoff frequency (*f*_C_), such as heart sounds (*f*_C_: ~500 Hz).

**Quantification of sound pressure level.** Pressure level of sounds were quantified by measuring A-weighted sound pressure level (SPL) using SPL meter function implemented in MATLAB^7,8^. Frequency spectra of acquired sounds were estimated via the Discrete Fast Fourier Transform (DFT) with A-weighting filter. Time-weighted sound level, defined as the ratio of the time-weighted root mean squared sound pressure to the reference sound pressure, was determined. Specifically, DFT of acquired sounds was obtained as follows:

$\text{X}\left[ \text{k} \right]\text{ = }\sum_{\text{N}\text{=0}}^{\text{N}\text{-1}} \text{x}\text{[}\text{n}\text{]}\text{e}^{\text{-}\text{j}\text{2}\text{π}\frac{\text{nk}}{\text{N}}}$ [2]

where *x*[*n*] is a discrete input signal with N samples and X[*k*] is the DFT of the input signal. Notably, DFT provides information about the frequency distribution of input signals with no temporal resolution while STFT described above provides both temporal and frequency resolution. The A-weighting filter (*H*_A_(*f*)) is defined as^9–11^:

$$H_{A}\text{(}f\text{)}\text{ = }\frac{(2{\text{π}f_{4})}^{2}{\cdot f}^{4}}{(f+2{\text{π}f_{1})}^{2}\cdot(f+2\text{π}f_{2})\cdot(f+2\text{π}f_{3})\cdot(f+2{\text{π}f_{4})}^{2}} [3]$$

where *f*_1_ = 20.6 Hz, *f*_2_ = 107.7 Hz, *f*_3_ = 737.9 Hz, and *f*_4_ = 12194.0 Hz. The weight functions and values are specified in the International Electrotechnical Commission (IEC) 61672 which is the current international standard that specifies sound level meter functionality and performance. The A-weighted DFT samples (*X*_A_[*k*]) are then calculated from equations 2 and 3 as:

$X_{A}\left[ \text{k} \right]\text{ = }H_{A}(f_{k})X\left[ \text{k} \right]\text{ }$ [4]

where *f_k_* represents the frequencies of each DFT sample *X*[*k*].

Finally, time-weighted sound level (*L*_t_), that is equivalent to the sound pressure level (SPL) reported in the manuscript, is determined as:

$\text{ }L_{t}\text{= }\text{10}{log}_{10}\left\{ \frac{h(y^{2})}{P_{0}^{2}} \right\}$ [5]

where y is the output of the A-weighting filter, *P*_0_ is a reference pressure defined as the lowest threshold of human hearing (20 μPa), and *h*(*y*^2^) represents the convolution of *y*^2^ with a filter of impulse response $\left( \frac{1}{\tau} \right)e^{\frac{-t}{\tau}}$ corresponding to a lowpass filter of the form of:

$H\left( s \right)=\frac{\frac{1}{\tau}}{s+\frac{1}{\tau}}$ [6]

The Laplace transfer function *H*(s) was converted into its discrete equivalent *H*(z) using the impulse invariant transform^12,13^:

$H\left( z \right)=\frac{\frac{1}{(\tau\times f_{s})}}{1-e^{\frac{-1}{(\tau\times f_{s})}}z^{-1}}$ [7]

where *f*_s_ is the audio sample rate and τ is the time-weighting coefficient (i.e., τ = 0.125 for fast time weighting, τ = 1 for slow time weighting). In this study, all *f*_s_ was 44.1 kHz and τ used for all sound level evaluation was 0.125. Calculated values of the time-weighted level of A-weighted sound pressure measured were quantified in “A-weighted decibels” (dBA).

**Assessment of correlation between the airway pressure and sound pressure level.** To quantify association between airway pressure (*P_airway_*) and A-weighted sound pressure level (SPL) acquired from both rat and swing lungs with air leak, we performed the correlation analysis using the time series of the measured data. To do this, all datasets were normalized via the following equation^14^:

$x^{'}=\frac{(x-x_{min})}{(x_{max}-x_{min})}$ [8]

where *x*’ is the normalized data, *x* is the original data, *x_min_* is the minimum value in the data set, and *x_max_* is the maximum value in the data set. Then, Pearson correlation coefficients (ρ) for rat and swine were calculated to determine correlation between the sound intensity and airway pressure (i.e., *P_airway_* , SPL)^15,16^.

$\rho\left( A,B \right)= \frac{1}{N-1}\sum_{i=1}^{N} \left( \frac{A_{i}-\mu_{A}}{\sigma_{A}} \right)\left( \frac{B_{i}-\mu_{B}}{\sigma_{B}} \right)$ [9]

where *μ_Α_* and *σ_Α_* are the mean and standard deviation of a data set *A*, respectively, and *μ_B_* and *σ_B_* are the mean and standard deviation of a data set *B*.

**Generation of sound intensity map.** The heatmap of the sound intensity for rat and swine lungs were obtained by measuring an average SPL value at different locations across the surface of the lung. Acoustic scanning was performed by positioning the microphone 1 cm from the lung pleural surface at the point of interest (i.e., upper left corner). At each point of interest, sounds were recorded for 5 seconds and average sound pressure level was calculated using MATLAB immediately after the sound recording. Once measurement was completed at a point, the microphone was moved by 1 cm for rat lungs and 2 cm for swine lung to an adjacent point for sound intensity quantification across a specified area of the lung. The total acoustic scan area for rat lungs and swine lungs were 5 cm × 5 cm and 6 cm × 8 cm, respectively. The average SPL values obtained within the scanned areas were normalized against the maximum SPL value recorded from each lung, which was consistently obtained just above the air leak site. This normalization resulted in the SPL values ranging between 0 and 1 that were used to create sound intensity maps. In the maps, the maximum SPL is indicated by “red” and the minimum SPL is indicated by “white” while intermediate values are interpolated between these two colors. To facilitate visualization of the sound intensity at the measurement area, the sound intensity map and a photograph of corresponding lung region were overlaid.

**Statistical tests.** All data were obtained from experiments repeated at least three times. All data analyses were conducted in Microsoft Excel, R, and GraphPad (Prism). Results are reported as the mean and standard deviation of measured values. One-way analysis of variance (ANOVA) was used to determine statistically significant differences between groups, with *p* < 0.05 considered significant.

**III. Supplementary Figures and Tables**

Supplementary Fig. 1 | Progression of pulmonary air leak to pneumothorax and tension pneumothorax. a, Pulmonary air leak is a common complication of lung surgery and occurs when air leaks from the airspace in the lung into the pleural space. b, Pneumothorax occurs when intrapleural pressure increases causing lung to collapse. c, Tension pneumothorax occurs when intrapleural pressure increases significantly, causing the mediastinum to shift and impairing venous return. Tension pneumothorax can cause hemodynamic instability, obstructive shock, and death.

Supplementary Fig. 2 | Generation of air leak sounds. Air leak sounds are generated as air escapes through a defect in the visceral pleura of the lung. Kinetic energy is transferred from the escaping air to the surrounding tissue, vibrating the tissue at distinct frequencies. When airflow-induced tissue vibration produces sounds that exceed sound pressure ~20 μPa, audible sounds are generated.

Supplementary Fig. 3 | Setup of pulmonary air leak sound analysis system. a, Schematic of computer-assisted sound analysis system. DAQ: data acquisition system. S: pressure and flow sensors. b, Photograph of pulmonary air leak sound analysis system. Inset: Microphone used for air leak sound acquisition.

Supplementary Fig. 4 | Rat pulmonary air leak model. a, Photograph of setup used to monitor air pressure in rat lungs. b, Photograph of air leak in ventilated rat lungs. c, Pressure inside the lung airway (*P*_Airway_) was measured while a focal puncture injury was induced using 18-gauge (18G) needle. Insp: inspiration. Exp: expiration. d, Air pressure curves obtained from control rat lung and lung punctured with 18G needle. e, Pressure curve obtained from lungs injured using a 16-gauge (16G) needle. f, Air pressure curves obtained 1 second after injury with 16G needle and 15 seconds post-injury.


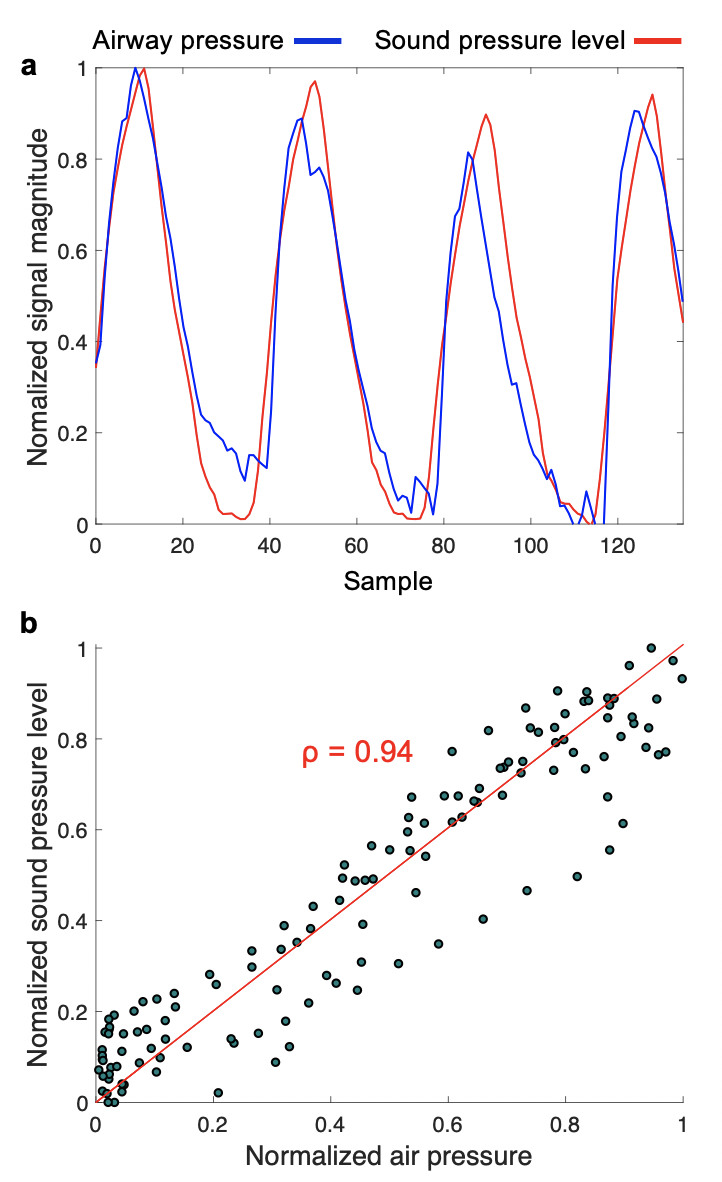


**Supplementary Fig. 5 | Evaluation of the correlation between airway pressure and sound pressure level in rat lungs with air leak**. **a**, Normalized magnitudes (range between 0 and 1) of airway pressure (*P_airway_*) and A-weighted sound pressure level (SPL) displayed in a single plot. **b**, Scatter plot showing correlation between normalized *P_airway_* and SPL where the relation is roughly linear as the dots are distributed along a straight line. Pearson correlation coefficient (ρ) was determined to be 0.94, suggesting a strong positive correlation between these two data sets.

Supplementary Fig. 6 | Spectral analysis of pulmonary air leak sounds in rat model. a, Spectrogram of air leak sound during one breathing cycle with discernible frequency bands (fb1 – fb7). b, Power spectrum of the signal within the dotted region in (a), obtained using Fourier transform.

Supplementary Fig. 7 | Swine pulmonary air leak model. a, Photograph of wedge resection performed on right middle lobe of swine lung using surgical stapler. b, Photograph of representative resected lung wedge. c, Photograph of staples (~30% of total) removed from the staple line to induce pulmonary air leak. d, Change in tidal volume due to pulmonary air leak (*n* = 4 swine). Mean loss in tidal volume: 27.4%. * *p* = 0.003. e, Application of sound analysis system to intraoperatively assess pulmonary air leak.


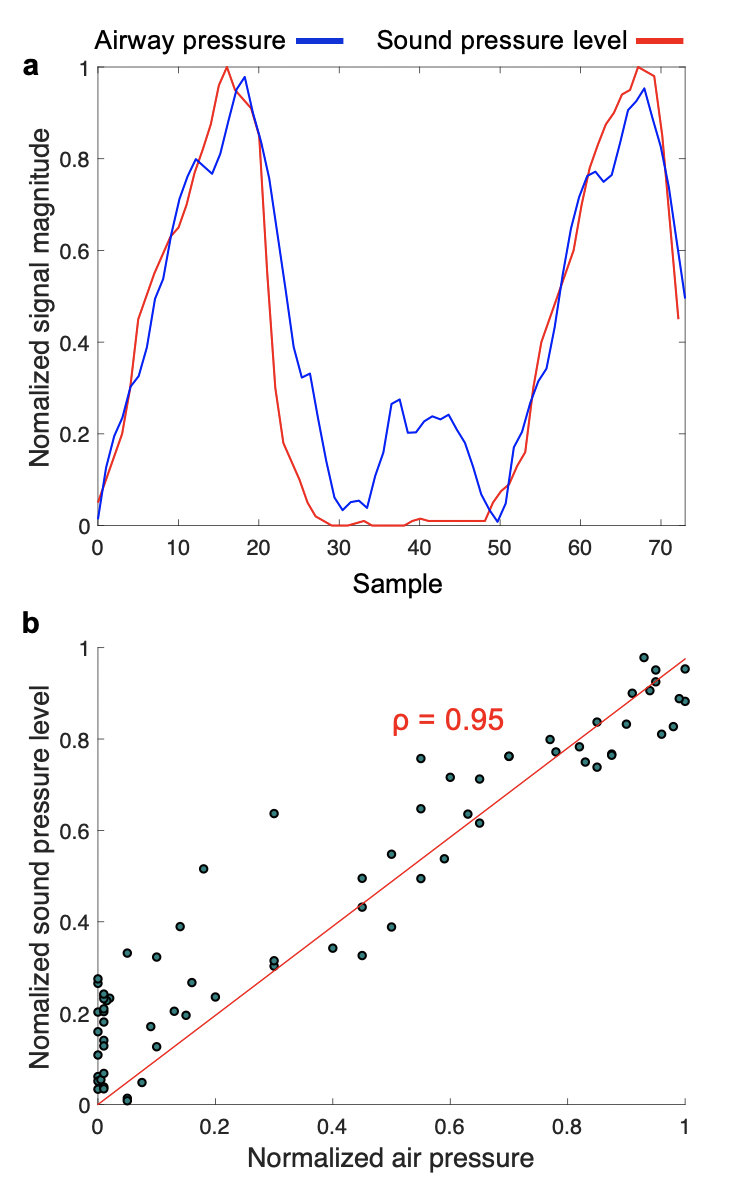


Supplementary Fig. 8 | Evaluation of the correlation between the airway pressure and sound pressure level in swine lung with air leak. a, Normalized magnitudes (range between 0 and 1) of airway pressure (*P_airway_*) and A-weighted sound pressure level (SPL). b, Scatter plot showing linear correlation between normalized *P_airway_* and SPL. Pearson correlation coefficient (ρ) was determined to be 0.95, suggesting a large positive correlation.


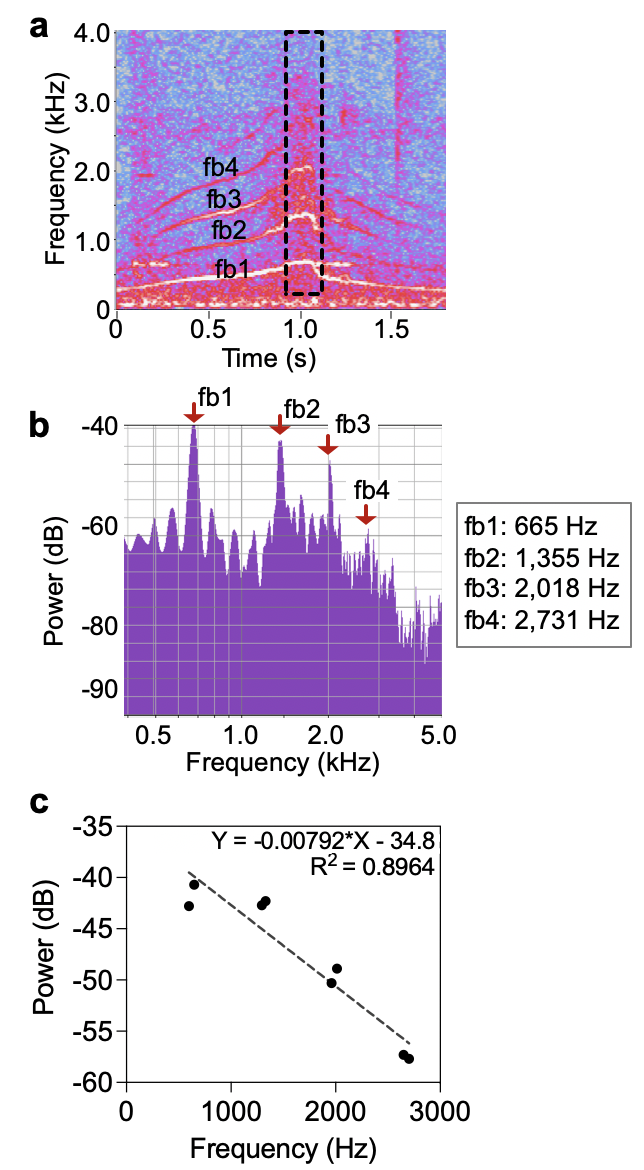


**Supplementary Fig. 9 | Spectral analysis of pulmonary air leak sounds in swine model.** **a**, Spectrogram of the air leak sound during one breathing cycle with discernible frequency bands (fb1 – fb4). **b**, Power spectrum of the signal within the dotted region in (a), obtained using Fourier transform. **c**, Relationship between band frequency and sound intensity (*p* < 0.001). Y = -0.00792X – 34.77, where X is frequency and Y is sound band intensity.

Supplementary Fig. 10 | Spectral analysis of pulmonary air leak sounds in swine model. a, Spectrogram of baseline with no air leak and thus no tidal volume loss (*TV*_Loss_: 0 mL/breath). b, Spectrogram of mild air leak with tidal volume loss of 8 mL/breath. c, Spectrogram of moderate air leak with tidal volume loss of 46 mL/breath. *TV*_Loss_: tidal volume loss.


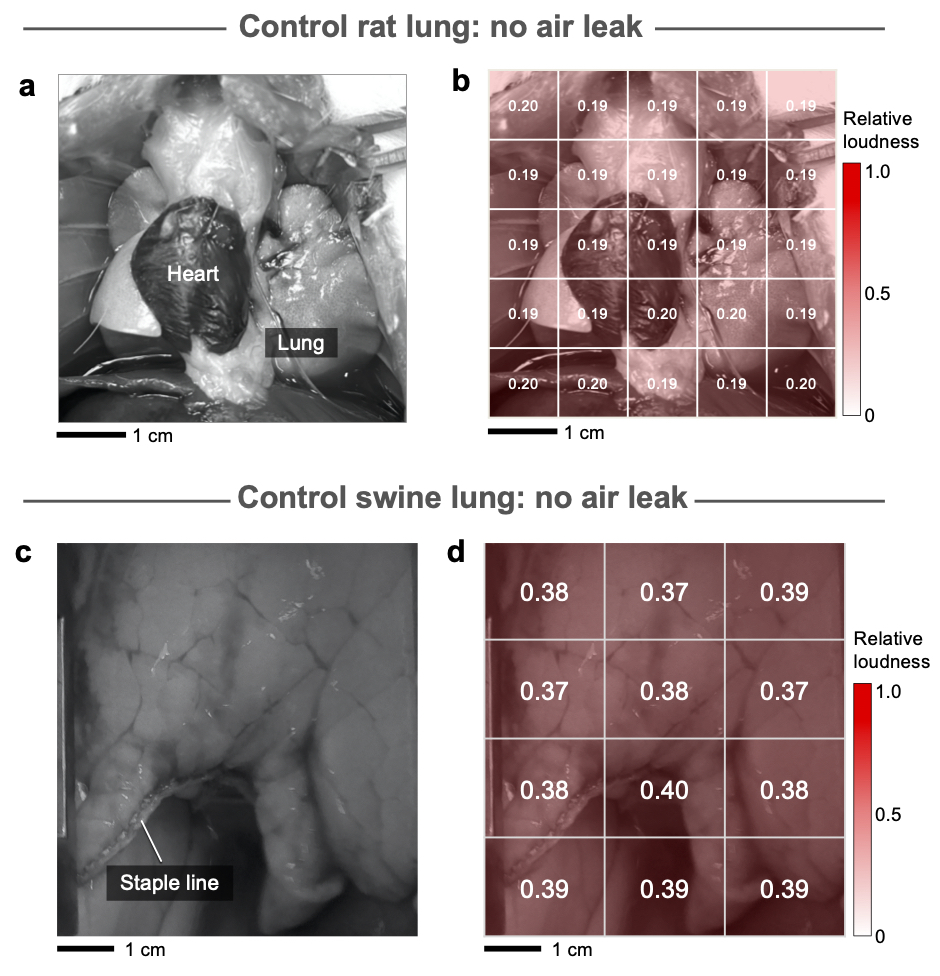


Supplementary Fig. 11 | Measurement of relative loudness in control lungs with no air leak. a, Photograph of control rat lung with no air leak and b, corresponding sound intensity heat map. c, Photograph of control swine lung right middle lobe with no air leak and d, corresponding sound intensity heat map.

Supplementary Fig. 12 | Spectral analysis of mixed air leak and heart sounds. a, Generation of mixed sound by mixing recorded human heart sounds and swine air leak sounds b, Spectrogram of heart sounds recorded in a healthy human subject. S1: sound 1. S2: sound 2. c, Spectrogram of mixed air leak and heart sounds. d, Amplitude of mixed air leak and heart sounds.

Supplementary Fig. 13 | Extraction of air leak sounds from a mixed sound. a, Histogram of power spectral density of the mixed sound (air leak and heart sounds: blue) and filtered sound (pink). Filtering of heart sounds was achieved via high-pass filtering. b, Spectrogram of the filtered sound. c, Filtered amplitude to obtain isolated air leak sound.


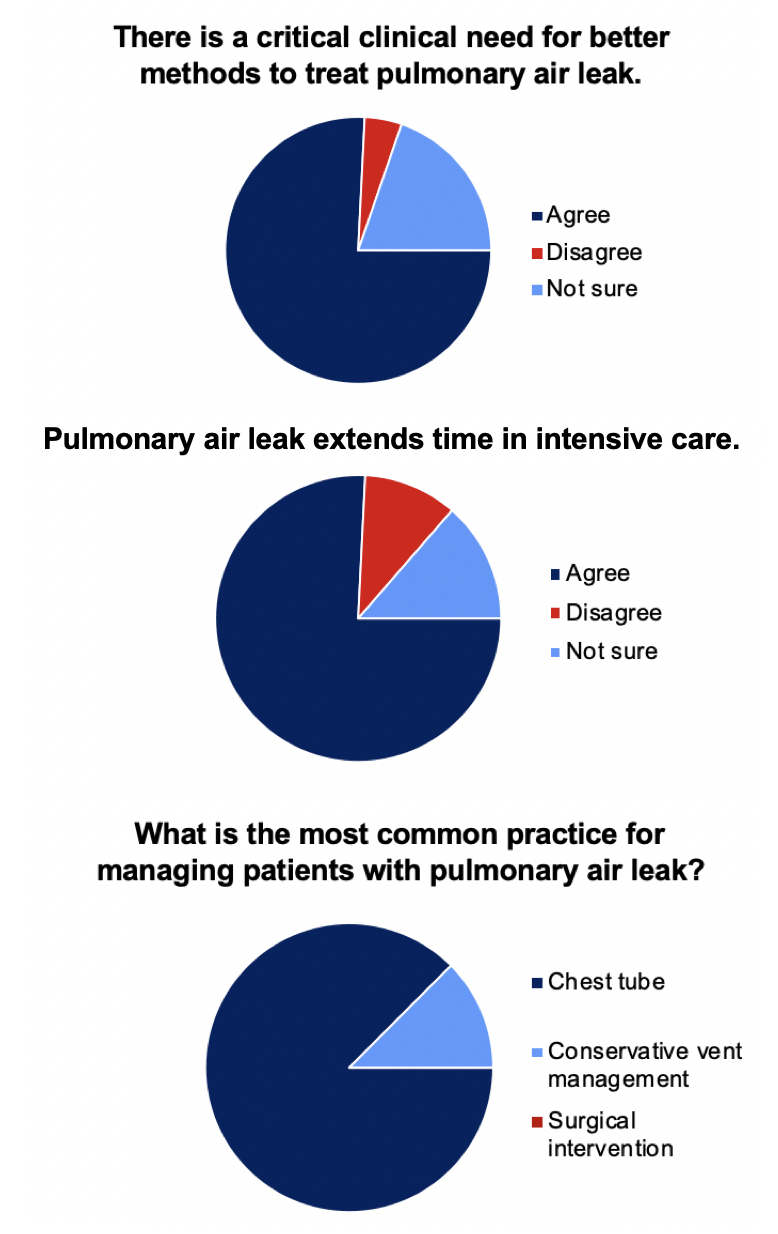


**Supplementary Fig. 14 |** **Results from survey of healthcare providers (n=66) treating patients with pulmonary air leak.** Survey conducted from 5 June 2020 – 15 July 2020. Survey population included physicians (n=47), nurses (n=17), nurse practitioner (n=1), and physician’s assistant (n=1) across thoracic surgery, general surgery, emergency medicine, and critical care.


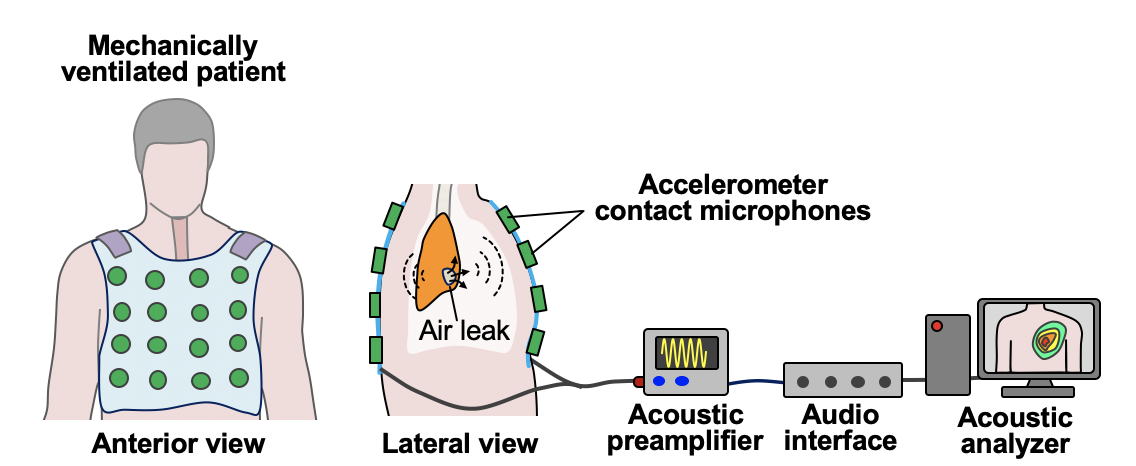


**Supplementary Fig. 15 | Schematic of envisioned non-invasive transthoracic air leak sound analysis system.** Patient with pulmonary air leak wearing transthoracic accelerometer contact microphone array. Acoustic signals are collected by accelerometer contact microphone sensors, amplified by acoustic preamplifier, and conveyed by audio interface to a computer for acoustic signal processing including high pass and adaptive filtering for quantitative assessment and localization of air leak.


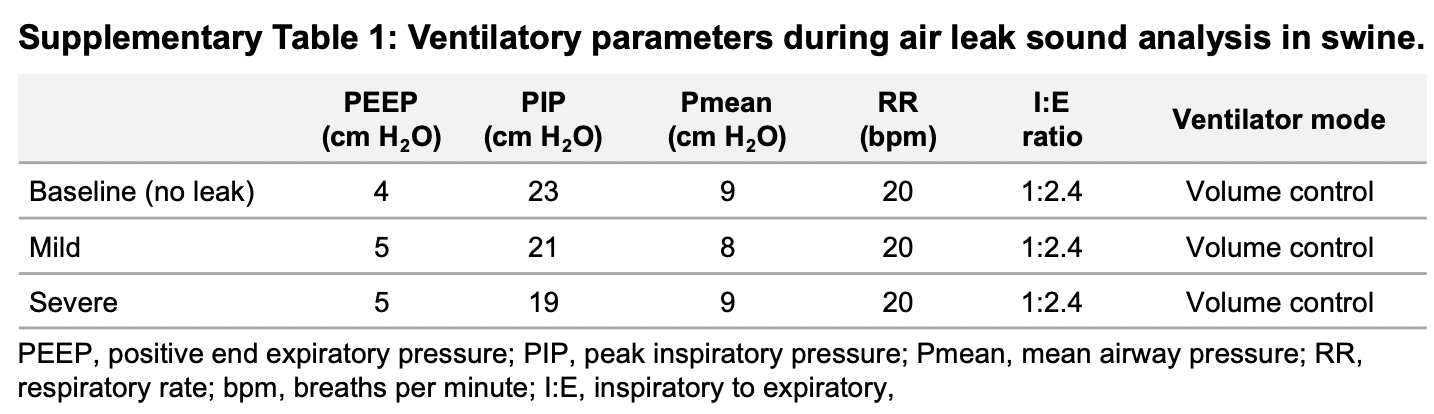


Supplementary Table 2 | Robert David Cerfolio (RDC) classification system for air leaks with possible causes, severity, and prevalence of each type of air leak^17–19^.


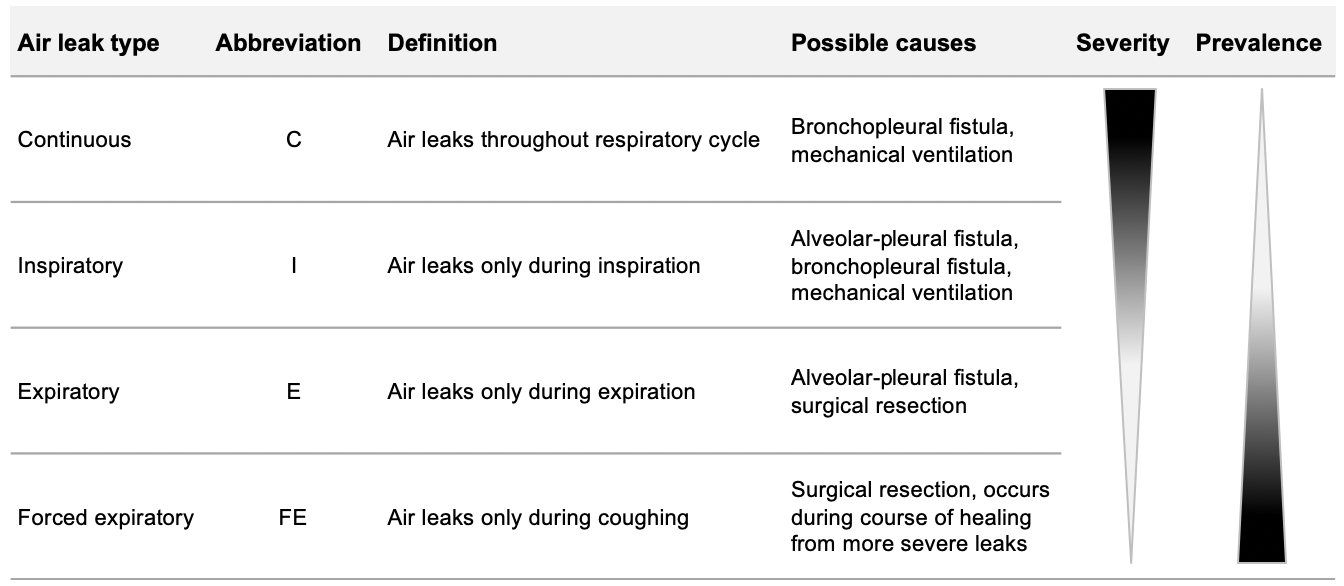


Supplementary Table 3 | Clinical modalities for detection of air leak^20–25^.

Supplementary Table 4 | Clinical modalities for intraoperative detection of air leak.^26–28^


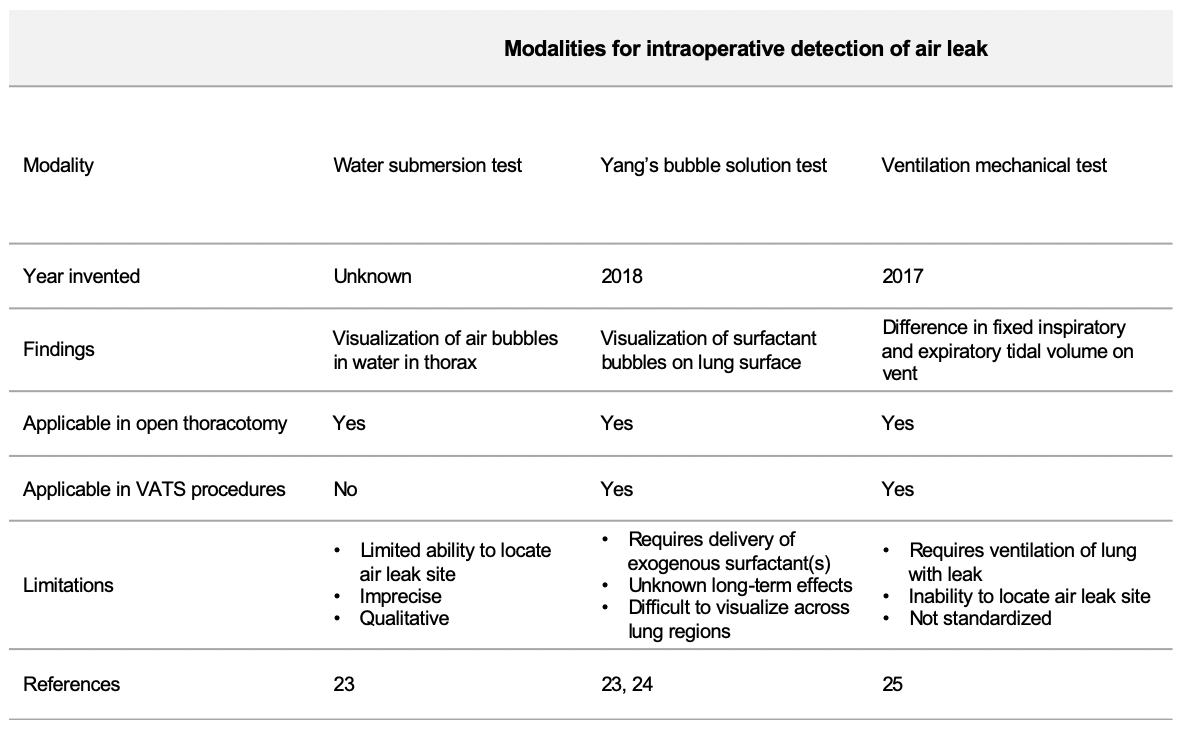


Supplementary Table 5 | Clinical modalities for detection of pneumothorax^29–36^.


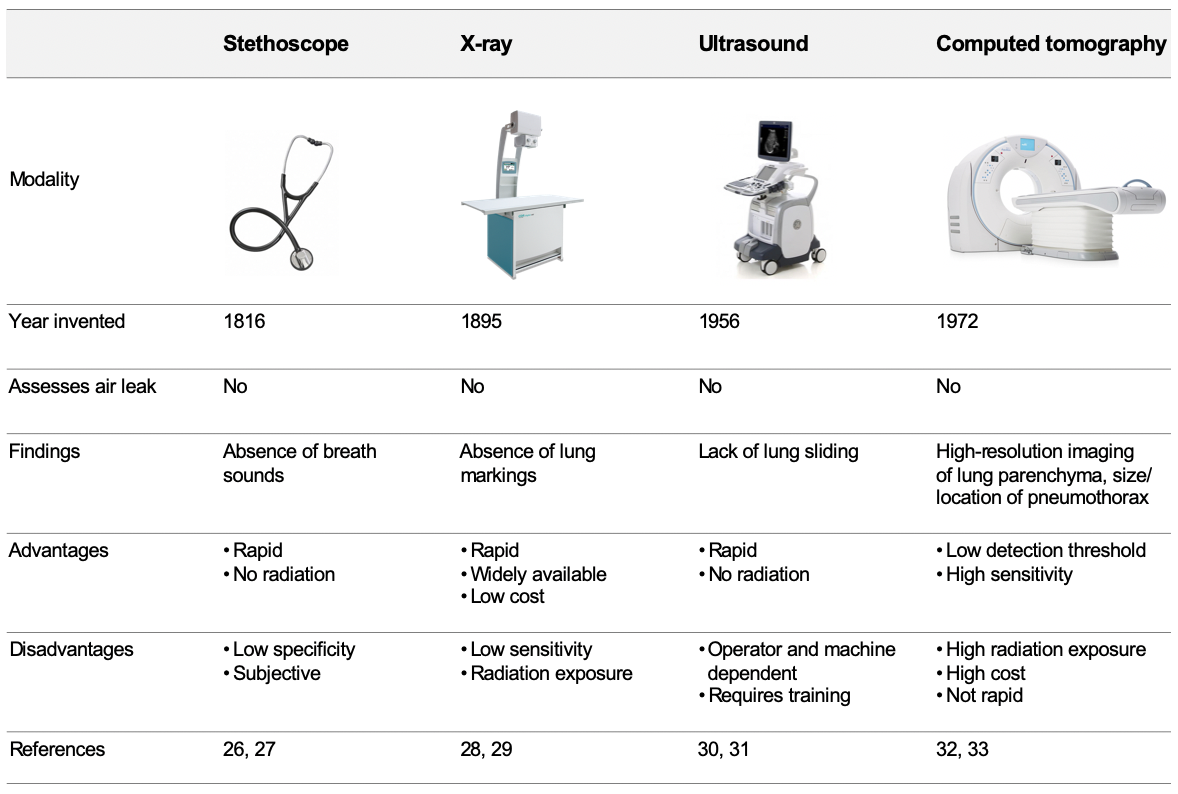


Supplementary Table 6 | Envisioned clinical applications for quantitative auscultation^37,38^


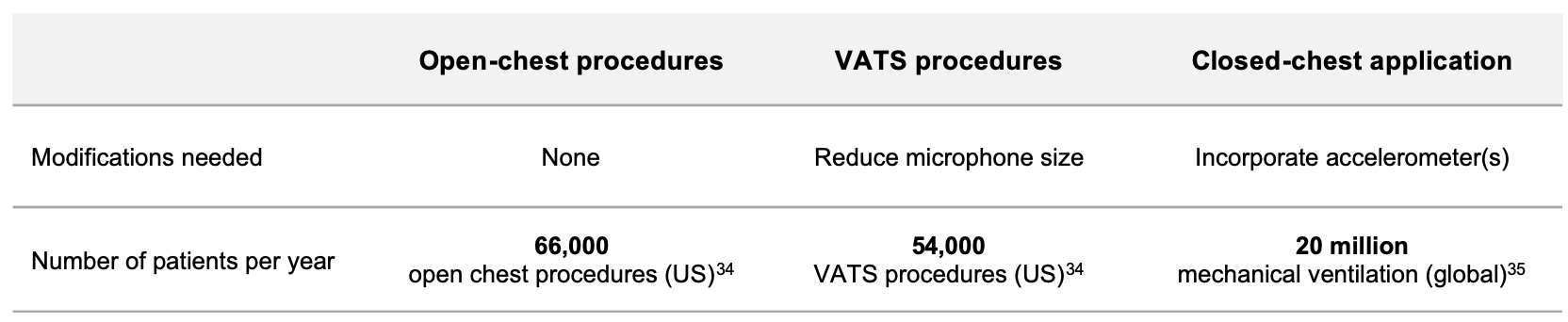


IV. Supplementary Video Captions

Supplementary Video 1 | Induction of pulmonary air leak in rat through focal puncture with 18-gauge needle.

**Supplementary Video 2 |** Rat model of pulmonary air leak.

**Supplementary Video 3 |** Air leak sounds from rat model of pulmonary air leak.

**Supplementary Video 4 |** Swine model of pulmonary air leak. Lungs with air leak were submerged in saline to visualize resultant air bubbles.

**Supplementary Video 5 |** Air leak sounds from swine model of pulmonary air leak.

**Supplementary Video 6 |** Air leak sounds recorded in swine with mild pulmonary air leak.

**Supplementary Video 7 |** Air leak sounds recorded in swine with severe pulmonary air leak.

**Supplementary Video 8 |** Sounds recorded in swine with normal lungs (i.e., no air leak).

**Supplementary Video 9 |** Mixed healthy human heart and swine pulmonary air leak sounds.

**Supplementary Video 10 |** Air leak sounds filtered from mixed human heart and swine pulmonary air leak sounds.

**V. Supplementary References**

1. Grasso S, Stripoli T, De Michele M, et al. ARDSnet Ventilatory Protocol and Alveolar Hyperinflation. *Am J Respir Crit Care Med*. 2007;176(8):761-767. doi:10.1164/rccm.200702-193OC

2. Burgess JK, Mauad T, Tjin G, Karlsson JC, Westergren-Thorsson G. The extracellular matrix – the under-recognized element in lung disease? *The Journal of Pathology*. 2016;240(4):397-409. doi:10.1002/path.4808

3. Zhivomirov H. On the Development of STFT-analysis and ISTFT-synthesis Routines and their Practical Implementation. *undefined*. Published online 2019. Accessed June 9, 2021. /paper/On-the-Development-of-STFT-analysis-and-Routines-Zhivomirov/ff5fc158123f90b9e57860d373378340320fb60c

4. Griffin D, Jae Lim. Signal estimation from modified short-time Fourier transform. In: *ICASSP ’83. IEEE International Conference on Acoustics, Speech, and Signal Processing*. Vol 8. Institute of Electrical and Electronics Engineers; 1983:804-807. doi:10.1109/ICASSP.1983.1172092

5. Suzuki A, Sumi C, Nakayama K, Mori M. Real-time adaptive cancelling of ambient noise in lung sound measurement. *Med Biol Eng Comput*. 1995;33(5):704-708. doi:10.1007/BF02510790

6. Emmanouilidou D, McCollum ED, Park DE, Elhilali M. Adaptive Noise Suppression of Pediatric Lung Auscultations With Real Applications to Noisy Clinical Settings in Developing Countries. *IEEE Trans Biomed Eng*. 2015;62(9):2279-2288. doi:10.1109/TBME.2015.2422698

7. Sadagopan N, Huber JE. Effects of Loudness Cues on Respiration in Individuals with Parkinson’s disease. *Mov Disord*. 2007;22(5):651-659. doi:10.1002/mds.21375

8. Huber JE. Effect of Cues to Increase Sound Pressure Level on Respiratory Kinematic Patterns during Connected Speech. *J Speech Lang Hear Res*. 2007;50(3):621-634. doi:10.1044/1092-4388(2007/044)

9. RIMELL AN, MANSFIELD NJ, PADDAN GS. Design of digital filters for frequency weightings (A and C) required for risk assessments of workers exposed to noise. *Ind Health*. 2015;53(1):21-27. doi:10.2486/indhealth.2013-0003

10. Harris C, Tocci G. Handbook of Acoustical Measurements and Noise Control, 3rd edition. Published online 1993. doi:10.1121/1.405398

11. A. A. S.-P. 1. American National Standard Electroacoustics–Sound Level Meters–Part 1: Specifications (a nationally adopted international standard). Published online 2014.

12. Smith JO. *Introduction to Digital Filters: With Audio Applications*. 2. print.; 2008.

13. Oppenheim AV, Schafer RW, Buck JR. *Discrete-Time Signal Processing*. 2nd ed. Prentice Hall; 1999.

14. Fisher RA. *Statistical Methods for Research Workers. Thirteen Edition, Revised.* Hafner; 1958.

15. Kendall MG, Stuart A. *Inference and Relationship*. Macmillan; 1979.

16. Press WH, Flannery BP, Teukolsky SA, Vetterling WT. *Numerical Recipes in C: Diskettes IBM 3 1/2" for IBM PC, PS/2, DOS*. Cambridge Univ. Press; 1992.

17. Cerfolio RJ, Tummala RP, Holman WL, et al. A prospective algorithm for the management of air leaks after pulmonary resection. *The Annals of Thoracic Surgery*. 1998;66(5):1726-1730. doi:10.1016/S0003-4975(98)00958-8

18. Cerfolio RJ. Advances in thoracostomy tube management. *Surg Clin North Am*. 2002;82(4):833-848, vii. doi:10.1016/s0039-6109(02)00026-9

19. Cerfolio RJ, Bass C, Katholi CR. Prospective randomized trial compares suction versus water seal for air leaks. *Ann Thorac Surg*. 2001;71(5):1613-1617. doi:10.1016/s0003-4975(01)02474-2

20. Rodríguez M, Jiménez MF, Hernández MTG, Novoa NM, Aranda JL, Varela G. Usefulness of conventional pleural drainage systems to predict the occurrence of prolonged air leak after anatomical pulmonary resection. *Eur J Cardiothorac Surg*. 2015;48(4):612-615. doi:10.1093/ejcts/ezu470

21. Oh SG, Jung Y, Jheon S, et al. Postoperative air leak grading is useful to predict prolonged air leak after pulmonary lobectomy. *J Cardiothorac Surg*. 2017;12(1):1. doi:10.1186/s13019-017-0568-6

22. Yeung C, Ghazel M, French D, et al. Forecasting pulmonary air leak duration following lung surgery using transpleural airflow data from a digital pleural drainage device. *J Thorac Dis*. 2018;10(S32):S3747-S3754. doi:10.21037/jtd.2018.08.11

23. Takamochi K, Nojiri S, Oh S, et al. Comparison of digital and traditional thoracic drainage systems for postoperative chest tube management after pulmonary resection: A prospective randomized trial. *J Thorac Cardiovasc Surg*. 2018;155(4):1834-1840. doi:10.1016/j.jtcvs.2017.09.145

24. Nielsen KR, Blake LM, Mark JB, DeCampli W, McDougall IR. Localization of bronchopleural fistula using ventilation scintigraphy. *J Nucl Med*. 1994;35(5):867-869.

25. Roberts DA, Rizi RR, Lipson DA, et al. Detection and localization of pulmonary air leaks using laser-polarized 3He MRI. *Magnetic Resonance in Medicine*. 2000;44(3):379-382. doi:https://doi.org/10.1002/1522-2594(200009)44:3<379::AID-MRM6>3.0.CO;2-4

26. Yang HC, Chang HY. Novel air leak test using surfactant for lung surgery. *J Thorac Dis*. 2018;10(12):6472-6474. doi:10.21037/jtd.2018.11.34

27. Kawai H. Problems with using the air leak test with Yang’s bubble solution during video-assisted thoracic surgery. *J Thorac Dis*. 2019;11(3):630-631. doi:10.21037/jtd.2019.02.44

28. Zaraca F, Vaccarili M, Zaccagna G, et al. Can a standardised Ventilation Mechanical Test for quantitative intraoperative air leak grading reduce the length of hospital stay after video-assisted thoracoscopic surgery lobectomy? *J Vis Surg*. 2017;3:179-179. doi:10.21037/jovs.2017.11.02

29. Chen SC, Chang KJ, Hsu CY. Accuracy of Auscultation in the Detection of Haemopneumothorax. *European Journal of Surgery*. 1998;164(9):643-645. doi:10.1080/110241598750005516

30. Arts L, Lim EHT, van de Ven PM, Heunks L, Tuinman PR. The diagnostic accuracy of lung auscultation in adult patients with acute pulmonary pathologies: a meta-analysis. *Sci Rep*. 2020;10(1):7347. doi:10.1038/s41598-020-64405-6

31. Chiles C, Ravin CE. Radiographic recognition of pneumothorax in the intensive care unit. *Critical Care Medicine*. 1986;14(8):677-680.

32. Glazer HS, Anderson DJ, Wilson BS, Molina PL, Sagel SS. Pneumothorax: appearance on lateral chest radiographs. *Radiology*. 1989;173(3):707-711. doi:10.1148/radiology.173.3.2813774

33. Soldati G, Testa A, Sher S, Pignataro G, La Sala M, Silveri NG. Occult Traumatic Pneumothorax: Diagnostic Accuracy of Lung Ultrasonography in the Emergency Department. *Chest*. 2008;133(1):204-211. doi:10.1378/chest.07-1595

34. Alrajhi K, Woo MY, Vaillancourt C. Test Characteristics of Ultrasonography for the Detection of Pneumothorax: A Systematic Review and Meta-analysis. *Chest*. 2012;141(3):703-708. doi:10.1378/chest.11-0131

35. Wall S, Federle M, Jeffrey R, Brett C. CT diagnosis of unsuspected pneumothorax after blunt abdominal trauma. *American Journal of Roentgenology*. 1983;141(5):919-921. doi:10.2214/ajr.141.5.919

36. Neff MA, Monk JSJ, Peters K, Nikhilesh A. Detection of Occult Pneumothoraces on Abdominal Computed Tomographic Scans in Trauma Patients. *Journal of Trauma and Acute Care Surgery*. 2000;49(2):281-285.

37. Cools-Lartigue J, Park BJ. Economic assessment in minimally invasive thoracic oncological surgery—USA experience. *Shanghai Chest*. 2018;2(0). Accessed November 20, 2021. https://shc.amegroups.com/article/view/4731

38. Adhikari NK, Fowler RA, Bhagwanjee S, Rubenfeld GD. Critical care and the global burden of critical illness in adults. *The Lancet*. 2010;376(9749):1339-1346. doi:10.1016/S0140-6736(10)60446-1
